# Fermi’s Golden Rule Rate Expression for Transitions Due to Nonadiabatic Derivative Couplings in the Adiabatic Basis

**DOI:** 10.1021/acs.jctc.4c00590

**Published:** 2025-02-13

**Authors:** Seogjoo J. Jang, Byeong Ki Min, Young Min Rhee

**Affiliations:** † Department of Chemistry and Biochemistry, Queens College, City University of New York, 65-30 Kissena Boulevard, Queens, New York, New York 11367, United States; ‡ Ph.D. Programs in Chemistry and Physics, Graduate Center of the City University of New York, New York, New York 10016, United States; § Department of Chemistry, Korea Advanced Institute of Science and Technology, Daejeon 34141, Korea

## Abstract

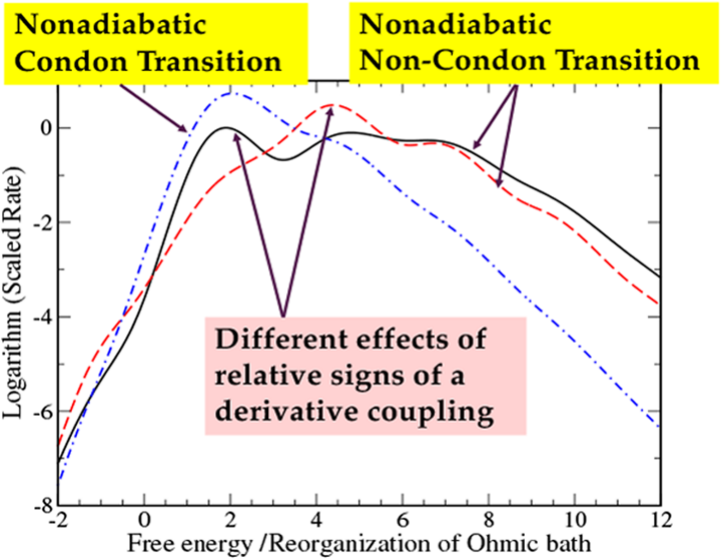

Starting from a general molecular Hamiltonian expressed in the basis of adiabatic electronic and nuclear position states, where a compact and complete expression for the nonadiabatic derivative coupling (NDC) Hamiltonian term is obtained, we provide a general analysis of the Fermi’s golden rule (FGR) rate expression for nonadiabatic transitions between adiabatic states. We then consider a quasi-adiabatic approximation that uses crude adiabatic states and NDC couplings, both evaluated at the minimum potential energy configuration of the initial adiabatic state, for the definition of the zeroth and first-order terms of the Hamiltonian. Although the application of this approximation is rather limited, it allows deriving a general FGR rate expression without further approximation while accounting for non-Condon contribution to the FGR rate arising from momentum operators of NDC terms and its coupling with vibronic displacements. For a generic and widely used model where all nuclear degrees of freedom and environmental effects are represented as linearly coupled harmonic oscillators, we derive a closed-form FGR rate expression that requires only Fourier transform. The resulting rate expression includes quadratic contributions of NDC terms and their couplings to Franck–Condon modes, which require evaluation of two additional bath spectral densities in addition to the conventional one that appears in a typical FGR rate theory based on the Condon approximation. Model calculations for the case where nuclear vibrations consist of both a sharp high-frequency mode and an Ohmic bath spectral density illustrate new features and implications of the rate expression. We then apply our theoretical expression to the nonradiative decay from the first excited singlet state of azulene, which illustrates the utility and implications of our theoretical results.

## Introduction

Advances in electronic structure calculation and quantum dynamics methods over past decades have made it possible to conduct first-principles dynamics calculation for many molecular systems.^1−10^ As yet, significant challenges remain for accurate quantum dynamics calculations of excited electronic states, especially for molecules in condensed or complex environments. One crucial issue that has to be addressed carefully in this regard is the fact that most quantum dynamics methods and rate theories have been developed under the assumption that it is possible to identify diabatic electronic states with constant or simple forms of electronic couplings between them. On the other hand, most quantum chemistry methods first seek the calculation of adiabatic electronic states for fixed nuclei, although alternative approaches are being developed and extended.^11−17^ In addition, accurately incorporating the effects of environmental dynamics into otherwise very high-quality *ab initio* calculations remains challenging.

In general, there is no genuine unitary transformation (independent of nuclear coordinates) from an arbitrary adiabatic basis to a true diabatic one. Thus, it remains an important practical issue to develop dynamics methods and rate theories starting from adiabatic states with as few assumptions as possible. To this end, full characterization of nonadiabatic derivative coupling (NDC) terms between adiabatic states is needed. In fact, theoretical works on this issue have a long history. Important formal developments have already been made.^18−30^ However, considering recent computational and experimental advances, it is meaningful to reassess issues addressed in earlier theoretical works in the context of modern computational modeling of nonadiabatic transitions.

While consideration in an adiabatic basis is straightforward for conducting nuclear quantum dynamics on a single adiabatic electronic surface, the extension of such an approach for electron–nuclear dynamics involving multiple adiabatic states, especially in condensed or complex environments, is challenging. To this end, various approximate methods^1−8,14,16,17,31−42^ have been developed. As yet, there are two important theoretical issues that need more careful theoretical consideration even for cases where the dynamics can be modeled as rate processes. One is the nonorthogonality of different adiabatic electronic states for different values of nuclear coordinates, and the other is the complicated nature of couplings between them. These issues are also important for the proper modeling of excitons formed in groups of molecules. Historically, Frenkel-type exciton-bath models^43−45^ have been used with reasonable success for describing many experimental data. However, the diabatic states used to define local site excitation states in these models are not always clearly defined.^46^ In addition, the extent of how and in what ways NDC terms contribute to the properties of excitons for many systems remain open issues.

In this work, we carefully consider NDC terms between adiabatic electronic states and provide a general FGR rate expression for nonadiabatic transitions between adiabatic states, which incorporates expressions used in many of earlier theories^18−21,24,25,27,28,47^ in a compact manner. Much of this amounts to a reformulation of already known theories but offers a new perspective. We then consider a well-defined Fermi’s golden rule (FGR) rate expression under a quasi-adiabatic approximation and provide a new closed-form FGR rate expression. This expression clearly accounts for non-Condon effects due to momentum contribution to the FGR rate expression and can be evaluated employing the data available from the standard *ab initio* and dynamics calculation for vibrational/environmental relaxation dynamics.

## Fermi’s Golden Rule Rate Expression for Nonadiabatic Transitions between Adiabatic States

Let us first provide a brief overview of the adiabatic states and nonadiabatic couplings. Although this is a standard topic of quantum chemistry, notations and definitions provided in this section are based on a more complete consideration^48^ and are different from conventional ones. In addition, the overview here will help clarify issues implicit in applying the standard FGR rate expression for nonadiabatic transitions and also provide a compact formalism for our rate expression. For a complete exposition, including textbook-level explanations, readers can refer to the Supporting Information (SI).

Consider a molecular system consisting of *N*_u_ nuclei, with their positions collectively represented by a 3N_u_ dimensional vector **R**, and assume that it is possible to identify two major adiabatic electronic states,^1^ |ψ_e,1_(**R**)⟩ and |ψ_e,2_ (**R**)⟩, which are well separated from other adiabatic electronic states. We assume that these two states have the same spin multiplicity and thus do not consider spin states explicitly here. Then, as detailed in the SI, the total molecular Hamiltonian in this subspace can be expressed as
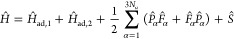
1where *Ĥ*_ad,1_ and *Ĥ*_ad,2_ are adiabatic components of the Hamiltonian, *P̂*_α_ is the one-dimensional (1D) nuclear momentum operator along the α direction, *F̂*_α_ is the operator representing the first nonadiabatic derivative coupling (NDC) terms, and *Ŝ* represents the second NDC terms. Note that the only approximation involved in eq 1 is the finite truncation of the electronic Hilbert space.

In more detail, the first two terms of eq 1, for *k* = 1, 2, are
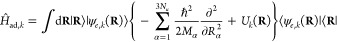
2where |**R**⟩ is the nuclear position state, *M*_α_ is the nuclear mass associated with the momentum along the α direction, and *U_k_*(**R**) is the sum of the eigenvalue *E_k_*(**R**) of the electronic state |ψ_*e,k*_(**R**)⟩ plus the nuclear potential energy terms. See SI for a more detailed expression for *U_k_*(**R**). Note that |**R**⟩|ψ_*e*,*k*_(**R**)⟩ (or ⟨ψ_*e*,*k*_(**R**)|⟨**R**|) is a short notation for a direct product form of electronic and nuclear states. This simple direct form involving an adiabatic electronic state is possible only for a nuclear position state for which an adiabatic electronic state is unambiguously defined.

As described (for a more general case) in the SI, the first NDC terms in eq 1 are expressed as
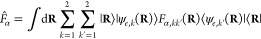
3with
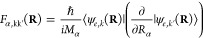
4and the second NDC term in eq 1 is expressed as
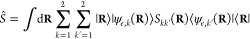
5with
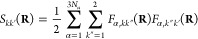
6The definitions given by eqs 4 and 6 make it clear that *Ĥ* defined by eq 1 is indeed Hermitian. It is also important to note that *S*_*kk*′_(**R**) contains only products of the first derivatives of adiabatic electronic eigenstates with respect to nuclear coordinates since second derivatives are already contained in *P̂*_α_*F̂*_α_ of eq 1.

For further simplification, let us assume that real-valued eigenfunctions for ⟨**r**|ψ_*e*,*k*_(**R**)⟩ can be identified, where **r** represents collectively positions of all of the electrons and ⟨**r**|the corresponding position bra. This assumption is valid for transitions involving singlet states in the absence of a magnetic field. Under this assumption, *F*_α,11_(**R**) = *F*_α,22_(**R**) = 0 and *S*_12_(**R**) = *S*_21_(**R**) = 0. For these cases, the second NDC terms are diagonal in the adiabatic basis and can be combined into the adiabatic terms of the Hamiltonian to define a zeroth-order Hamiltonian as follows:
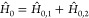
7where *Ĥ*_0,*k*_ = *Ĥ*_*ad*,*k*_ + *Ŝ*_*k*_ with
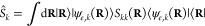
8Then, the full molecular Hamiltonian, eq 1, can also be expressed as
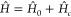
9where *Ĥ_c_* contains only the first NDC terms. This is off-diagonal with respect to adiabatic states and is given by
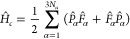
10

Having defined the zeroth-order Hamiltonian *Ĥ*_0_ and *Ĥ_c_*, eqs 7 and 10, it now seems straightforward to employ the following standard FGR rate expression:
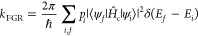
11where |ψ*_i_*⟩ and |ψ*_f_*⟩ are initial and final eigenstates of the zeroth-order Hamiltonian *Ĥ*_0_ with eigenvalues *E_i_* and *E_f_*, and *p_i_* is the probability for the initial state |ψ*_i_*⟩. However, the exact application of eq 11 still involves somewhat complicated theoretical issues, as detailed below.

Most theories^18−21,23−26^ of nonadiabatic transition have considered eq 11 as the starting point. However, with few exceptions,^22,29,30^ they used approximate expressions for the coupling obtained from nonadiabatic corrections of adiabatic vibronic wave functions. On the other hand, for proper application of eq 11, it is also important to identify the initial and final states that are genuine orthogonal eigenstates of *Ĥ*_0_ while being defined in the full direct product space of electronic and nuclear degrees of freedom. The issue at hand becomes clearer considering the following time domain expression for FGR:
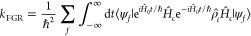
12where ρ̂*_i_* = ∑*_i_p_i_*|ψ*_i_*⟩⟨ψ*_i_*|. The above expression results directly from the first-order expansion of the propagator with respect to *Ĥ_c_* as follows:
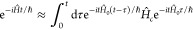
13and results in eq 11 only if *Ĥ*_0_|ψ*_i_*⟩ = *E_i_*|ψ*_i_*⟩ and *Ĥ*_0_|ψ*_f_*⟩ = *E_f_*|ψ*_f_*⟩. If not, eq 11 amounts to invoking an additional approximation.

In the SI, we prove directly that the following state indeed is an eigenstate of *Ĥ*_ad,*k*_:
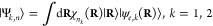
14where χ*_n_k__*(**R**) is a nuclear eigenfunction for the adiabatic potential energy, *U_k_*(**R**). Note that the projection of the above state onto a particular value of nuclear coordinate **R**′ results in ⟨**R**′|Ψ*_k,n_*⟩ = χ*_n_k__*(**R**′) |ψ_e,*k*_(**R**′)⟩, which is the conventional adiabatic electronic-nuclear wave function. As yet, |Ψ*_k,n_*⟩ is not the eigenstate of *Ĥ*_0_ unless *Ŝ* is constant, although this effect can be accounted for up to the first order by using an average value.

In the SI, we also provide a full expression for the matrix elements of *Ĥ_c_* with respect to states given by eq 14, which includes additional terms that have not been considered in earlier theories. These additional terms appear because eq 14 involves a linear combination of adiabatic electronic states that are not orthogonal to each other. Namely, it results from the fact that ⟨ψ*_k_*(**R**)|ψ_*k*′_ (**R**′)⟩ ≠ 0 for **R** ≠ **R**′ even for *k* ≠ *k*′, as long as they have the same spin symmetry. The expressions provided in the SI, eqs S20 and S21, also show that the effect of nuclear coordinate dependence of adiabatic states should be handled carefully including the second NDC terms as well.

The analysis provided above and the full expression for matrix elements of *Ĥ_c_* in the SI clarify issues concerning the exact calculation of the FGR rate for nonadiabatic transitions between adiabatic states. Errors due to making approximations in this calculation can be significant if adiabatic states change significantly with nuclear coordinates and/or *Ĥ_c_* is not small enough. Theories addressing these issues have indeed been developed but seem to have considered only parts of the matrix elements of *Ĥ_c_*.^22,23^ Alternatively, one can employ crude adiabatic states as the reference electronic states, which do not introduce complications resulting from the peculiar nature of adiabatic states. Indeed, advanced formulations in this direction, known as crude adiabatic schemes, have already been formulated.^29,30^ However, these have not yet been developed into practical computational methods to the best of our knowledge.

Our work here is focused on the case where *Ĥ_c_* remains small and changes modestly with nuclear coordinates. Thus, we here consider a quasi-adiabatic approximation at the level of the Hamiltonian operator, which replaces adiabatic electronic states involved in *Ĥ*_0_ and *Ĥ_c_*, eqs 7 and 10, with appropriate crude adiabatic states. This approximation accounts for the nonadiabatic coupling at the lowest order of the Hamiltonian and makes the application of FGR straightforward and well-defined. This also opens up future possibilities to make further theoretical advances following the idea of the crude adiabatic scheme^29,30^ and/or determining the correction terms of the Hamiltonian employing perturbative expansion^22^ of adiabatic states with respect to crude adiabatic states.

It is also interesting to note that our general FGR rate expression based on the simple quasi-adiabatic approximation turns out to be almost equivalent (save for the diagonal second NDC term) to the general expression used by Kubo and Toyozawa^19^ and that^25,26^ used later by Shuai and co-workers (without assumption of promoting mode), which were derived based on a Condon approximation for the first NDC terms. A similar expression was used for the calculation of nonradiative decay within the cumulant approximation by Borrelli and Peluso^28^ and more recently by Landi et al.^49^ As will become clear, what is new in our work is that we provide a convenient new form of the FGR rate expression applicable to processes in general environments, which can be calculated by incorporating standard *ab initio* data for excited states and dynamics data for molecular/environmental relaxation.

## Fermi’s Golden Rule Rate Expression Within a Quasi-Adiabatic Approximation

We here provide the FGR rate expression within the quasi-adiabatic approximation that uses the crude adiabatic states evaluated at the local minimum of **R** for the initial adiabatic Hamiltonian, *Ĥ*_ad,1_. A more detailed description is provided below. We also regard all other degrees of freedom except for the two electronic states 1 and 2 as the bath. Thus, the bath includes all of the molecular vibrations for isolated molecules and also additional environmental effects for molecules embedded in other media.

### General Expression

First, consider the case of an isolated molecule. Let us denote the minimum energy nuclear coordinates of *E*_*e*,1_(**R**) collectively as **R**_1_^g^. Then, we define the two adiabatic electronic states determined at **R**_1_^g^ as two electronic states independent of nuclear degrees of freedom as follows:
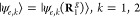
15It is assumed that these states serve as good approximations for adiabatic electronic states |ψ_*e*,*k*_(**R**)⟩ near the vicinity of **R**_1_^*g*^, where nonadiabatic transitions occur. Thus, the diagonal terms of the Hamiltonian in eq 9 are approximated as^48^
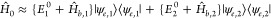
16For isolated molecules, *E*_*k*_^0^ = *U*_*k*_(**R**_1_^*g*^) + *S*_*kk*_(**R**_1_^*g*^) and
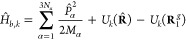
17Full expressions for *E*_*k*_^0^ + *Ĥ*_*b*,*k*_, both in terms of original nuclear coordinates and normal vibrational modes, are provided in the SI.

Similarly, it is assumed that the first NDC terms at **R**_1_^*g*^ also serve as a good approximation for those terms evaluated at nearby nuclear coordinates. Thus, the coupling term in eq 9 is approximated as
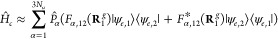
18where *F*_α,12_(**R**_1_^*g*^) is defined by eq 4 evaluated at **R**_1_^*g*^ for *k* = 1 and *k*′ = 2. In the above equation, the fact that *F*_α,21_(**R**_1_^*g*^) is the complex conjugate of *F*_α,12_(**R**_1_^g^) has also been used. Note also that *P̂*_α_ commutes with diabatic electronic states |ψ_*e*,1_⟩ and |ψ_*e*,2_⟩.

Note that *S*_11_(**R**_*g*_) = *S*_22_(**R**_*g*_) for the present case that has only two adiabatic states. Therefore, these second-order NDC terms are assumed not to affect the energy gap. In fact, the zeroth-order Hamiltonian, eq 16, can include the full second NDC term by replacing *U*_*k*_(**R̂**) with *U*_*k*_(**R̂**) + *S*_*kk*_(**R̂**) – *S*_*kk*_(**R**_1_^*g*^). Likewise, *F*_α,12_ in eq 18 and its Hermitian conjugate can be assumed to be functions of nuclear position operators such that each term in the summation is replaced with *P̂*_α_*F̂*_α,12_(**R̂**)|ψ_1_⟩ψ_2_|+ *F̂*_α,21_ (**R̂**)*P̂*_α_ |ψ_*e*,2_⟩⟨ψ_*e*,1_|. However, whether this more general assumption, without fully accounting for the nuclear coordinate dependence of adiabatic states, would consistently lead to an improvement is not yet clearly understood.

For the Hamiltonian terms *Ĥ*_0_ and *Ĥ*_*c*_ given by eqs 16 and 18, respectively, and for the following initial density operator:
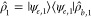
19with ρ̂_*b*,1_ = *e*^–β *Ĥ*_*b*,1_^/Tr{*e*^–β*Ĥ*_*b*,1_^}, it is now straightforward to apply FGR^50,51^ and obtain an expression similar to well-known expressions. To this end, it is convenient to use the mass-weighted coordinate system in the Eckart frame, followed by normal mode representation. A detailed mathematical procedure for this is reviewed in the SI.

So far, we have considered isolated molecules only, but all of the expressions can be extended to cases where molecules are embedded in some host environments, which are also detailed in the SI. The major change in this case is the extension of the bath Hamiltonians and the normal vibrational modes. Even in this case, the formal expression for *Ĥ*_*c*_ in terms of normal modes remains the same and is given by
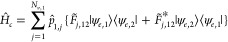
20with
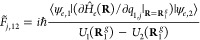
21where we have used the fact that *E*_1_ (**R**_1_^*g*^) – *E*_2_ (**R**_1_^g^) = *U*_1_(**R**_1_^g^) – *U*_2_(**R**_1_^g^).

In eq 20, *p̂*_1,*j*_ is the momentum operator for the *j*th normal mode defined with respect to the minimum potential energy structure of the adiabatic electronic state 1. As detailed in the SI, the above expression, for the case of isolated molecules, is obtained under the assumption that *Ĥ_c_* is independent of translation or rotation in the body fixed frame corresponding to the minimum energy structure for the adiabatic electronic state 1. For molecules embedded in other media, this can be justified in a more straightforward manner, as explained in the SI.

Given all of the definitions and assumptions, it is now straightforward to apply the time-dependent perturbation theory and obtain the following standard FGR rate expression:^51^
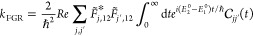
22where
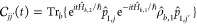
23with Tr*_b_* representing trace over all of the bath degrees of freedom.

Equation 22 with eq 23 is the most general expression based on the quasi-adiabatic approximation. It is interesting to note that this expression is equivalent to the general expression derived by Shuai and co-workers^25,26^ based on a Condon approximation for the first NDC term. In fact, a similar expression has been presented much earlier by Kubo and Toyozawa in their generating function formalism for radiative and nonradiative processes involving trapped electrons in crystal environments.^19^

Note that eq 22 is valid for any form of *Ĥ*_*b*,1_ and *Ĥ*_*b*,2_ and thus can be used to understand the effect of anharmonic terms in combination with appropriate quantum dynamics calculations. In fact, the total Hamiltonian defined here, *Ĥ*_0_ + *Ĥ_c_*, is amenable for the application of various quantum dynamics calculation methods currently available because |ψ_*e*,1_⟩ and |ψ_*e*,2_⟩ are diabatic states. Therefore, direct quantum dynamics calculations beyond the rate description are possible under the same quasi-adiabatic approximation as well.

### Closed-Form Expression for Linearly Coupled Harmonic Oscillator Bath

For the case where contributions of anharmonic terms are negligible, the bath Hamiltonians can be approximated as sums of harmonic oscillators as detailed in Sec. II of the SI. Let us consider the simplest case where all of the oscillators can be approximated as displaced ones, for which ω_*j*,2_ = ω_*j*,1_, *q̂*_2,*j*_ = *q̂*_1,*j*_+*d*_*j*_, and *p̂*_2,*j*_ = *p̂*_1,*j*_ in eq S49 of the SI. Without losing generality, we can drop the subscript 1 for *Ĥ*_*b*,1_ and ρ̂_*b*,1_ and introduce a bath operator *B̂* such that *Ĥ*_*b*,2_ = *Ĥ*_*b*_ + *B̂* + ∑_*j*_ ω_*j*_^2^*d*_*j*_^2^/2. Thus, *Ĥ*_0_ for this case can be expressed as
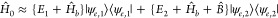
24where *E*_1_ = *E*_1_^0^, *E*_2_ = *E*_2_^0^ + ∑_*j*_ ω_*j*_^2^*d*_*j*_^2^/2, , and *B̂* = ∑_*j*_ ℏω_*j*_*g*_*j*_(*b̂*_*j*_ + *b̂*_*j*_^†^). In the above expressions, *b̂*_*j*_ and *b̂*_*j*_^†^ are the usual lowering and raising operators for harmonic oscillators. Thus, *b̂*_*j*_ = *x̂*_*j*_ + *ip̂*_*j*_/*b̂*_*j*_^†^ = *x̂*_*j*_ − *ip̂*_*j*_/ and *g*_*j*_ = *d*_*j*_ω_*j*_/ Then, the bath correlation function for the present case can be expressed as
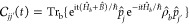
25The above bath correlation function can be evaluated explicitly as described in the Appendix, leading to the following closed-form expression for the FGR rate
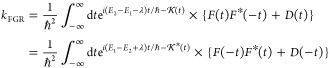
26where expressions for , *F*(*t*), and *D*(*t*) are given by eqs 74, 76, and 77, respectively. The second equality in the above equation is obtained by either taking a complex conjugate of the integrand or replacing *t* with – *t* of the first expression. Thus, . We provide this latter expression since it corresponds to a more conventional one and will be used for the numerical calculation.

Equation 26 is the major result of the present work. For large molecules or molecules embedded in other environments, the distribution of normal vibrational modes included in , *F*(*t*), and *D*(*t*) forms a (near) continuum. For this case, it is necessary to represent them in terms of the bath spectral densities. For , the following spectral density is well established:
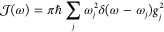
27For *D*(*t*) and *F*(*t*), we introduce two new spectral densities as follows:
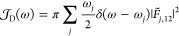
28
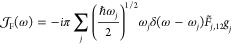
29Note that  is real-valued and positive as is .  which represents the sum of couplings between displacements of normal modes and the projections of the first NDC terms onto corresponding modes, is also real-valued because *F̃*_*j*,12_ is purely imaginary under the assumption that all of the adiabatic electronic wave functions in eq 21 are real-valued. However,  is not necessarily positive unlike  and  although it is still bounded in its magnitude as follows:
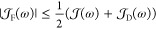
30Then, the bath correlation functions that appear in eq 26 can be expressed in terms of the spectral densities defined above as follows:
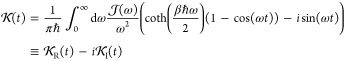
31
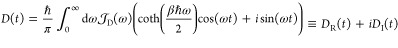
32
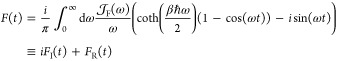
33

The above representations of time correlation functions in terms of bath spectral densities have significant implications both conceptually and practically. They retain all of the necessary information about the effects of molecular vibrations and environmental responses within the linear response approximation and can be employed to some extent even beyond the model of linearly coupled harmonic oscillator bath models. Determination of  from the energy gap correlation function^52,53^ of dynamics simulation is well established and has been confirmed to work well for the modeling of absorption spectra. For the present purpose and also for emission spectra,  can be calculated through dynamics simulation for the excited potential energy surface. For  and , similar approaches incorporating the information on NDC terms can also be developed.

## Model Calculations

We here consider a generic model for a molecule with one prominent vibrational frequency embedded in liquid or disordered solid environments, where low-frequency vibrational modes of molecules plus environmental effects are typically modeled well in terms of Ohmic bath spectral densities.^46,52,54−58^ Given that the spectral range of the Ohmic bath is smaller than the frequency of the molecular vibration, this situation can be modeled well^59,60^ by the following sum of Ohmic and delta function spectral densities:
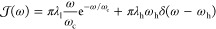
34where λ_l_ and λ_h_ are components of reorganization energies due to the low-frequency Ohmic part and the isolated high-frequency parts. The same model was considered^61^ for the generalization of the energy gap law.

For the present work, we also need to consider the forms of bath spectral densities for  and  as well. Since there are not sufficient physical or computational data for these spectral densities yet, we suppose similar forms for these as follows:
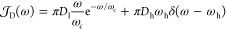
35
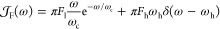
36where *D*_l_ and *D*_h_ represent squared magnitudes of NDC terms, whereas *F*_l_ and *F*_h_ correspond to sums of couplings between the NDC and vibronic displacement terms over all of the vibrational modes. These four parameters are all defined in units of energy. Alternatively, these can be expressed in terms of dimensionless parameters, η, *s*_h_, η__D__, *s*__D__, η__F__, and *s*__F__, as indicated in Table 1. Since the high-frequency delta function part originates from a specific molecular vibration, the magnitude of *s*__F__ is determined given the values of *s*_h_ and *s*__D__ such that *s*__F__^2^ = *s*_h_*s*__D__. On the other hand, the value of *F*_l_ is not fully determined except for the condition that |*F*_l_| ≤ *D*_l_/2, which results from eq 30.

**Table 1:** Relationship between Parameters Defining Bath Spectral Densities

λ_l_	λ_h_	*D*_l_	*D*_h_	*F*_l_	*F*_h_
ηℏω_c_	*s*_h_ℏω_h_	η__D__ℏω_c_	*s*__D__ℏω_h_	η__F__ℏω_c_	*s*__F__ℏω_h_

For the spectral density of eq 34, the real and imaginary parts of , as defined through the last line of eq 74, can be expressed as
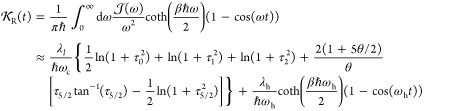
37
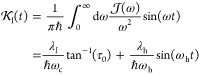
38where θ = βℏω_c_ and τ_n_ = ω_c_*t*/(1 + *nθ*). The second equality in eq 37 is based on the following approximation:^56^
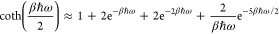
39and we have used an explicit expression for ∫_0_^τ_5/2_^ dτ′ tan^–1^ (τ′). On the other hand, the real and imaginary parts of *D*(*t*), which are defined by the last line of eq 77, are expressed as
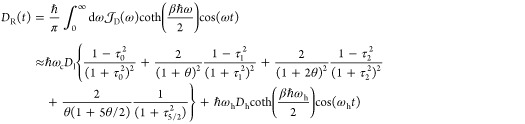
40
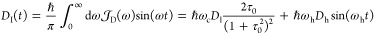
41Finally, the real and imaginary parts of *F*(*t*), which are defined by the last line of eq 76, have the following expressions
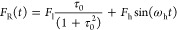
42
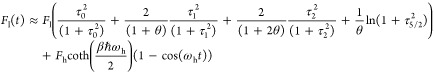
43Note that the second approximate equalities in eqs 40 and 43 are also based on the approximation of eq 39.

For numerical calculations, we considered two values of ω_h_/ω_c_ = 5 and 15, which respectively represent moderate and high-frequency vibrational frequencies. Given that ℏω_c_ ≈ 200 cm^–1^, the thermal energy at room temperature, the two choices of ω_h_ correspond to about 1000 and 3000 cm^–1^ for the vibrational frequencies. For each choice, we then considered three different cases of other parameters such that the sum of *D*_l_ and *D*_h_ remains the same. Case A represents the situation where NDC terms solely originate from the isolated vibrational mode. Cases B and C represent examples where the Ohmic bath parts make dominant contributions to NDC terms. In the presence of two different sources for NDC terms, the sign of *s*__F__ relative to η__F__ has different effects on rates. Cases B and C, respectively, correspond to the positive and negative signs of *s*_F_ while having the same values for all other parameters. In addition to *k*_FGR_ calculated exactly according to eq 26, we also calculated rates based on the following Condon approximation:
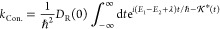
44The rate expression above employs NDC terms evaluated at *t* = 0 as effective Condon-coupling terms.

**Figure 1:**
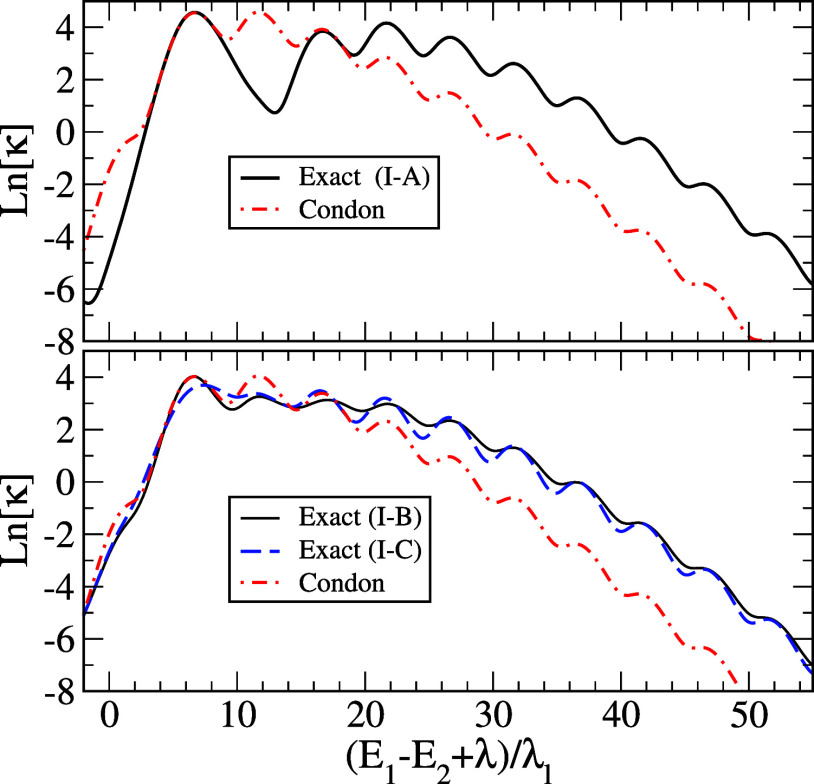
Natural logarithms of scaled rates  (in the units where *k*_B_ = ℏ = ω_c_ = 1) for case I-A (upper panel) and cases I-B and I-C (lower panel), are compared with reference results of a Condon approximation, eq 44, for the same cases.

Figure 1 shows results for cases I-A,B, and C of Table 2. The upper panel shows results for case I-A, and the lower panel compares the two results for cases I–B and I–C. The Condon approximations for these cases are the same, while slightly smaller than that for case I-A. The oscillatory behavior of rates with respect to the energy gap reflects the vibrational progression due to the single vibrational mode. It is clear that non-Condon effects due to momentum terms result in significant enhancement of rates as the energy gap increases when compared with the Condon approximation given by eq 44 in all cases.

**Table 2:** Table of Model Parameters for the Spectral Densities^a^

Case	ω_*h*_/ω_c_	*s*_h_	η__D__	η__F__	*s*__D__	*s*__F__
I-A	5	1	0	0	4	2
I-B	5	1	15	2	1	1
I-C	5	1	15	2	1	–1
II-A	15	0.2	0	0	1	
II-B	15	0.2	12	2	0.2	0.2
II-C	15	0.2	12	2	0.2	–0.2

a Two other parameters are set to *k*_BT_/(ℏω_c_) = 1 and η = 1. Note that *s*__F__^2^ = *s*_h_s_D_ and the result is independent of the sign of *s*__F__ for the case η__F__ = 0.

A comparison of the upper and lower panels of Figure 1 in the large energy gap limit shows that the rates for case A are consistently larger than those for cases B and C, which means that NDC terms coming from the single high-frequency vibrational mode are more effective in enhancing the rate compared with those from the lower frequency Ohmic part. Nonetheless, except for small or negative values of *E*_1_–*E*_2_, for which the validity of the quasi-adiabatic approximation may not be fully justified, the differences between the results for upper and lower panels are relatively small. The lower panel of Figure 1 shows that the case with negative *s*__F__ results in smaller rates than those for positive *s*__F__, as expected, but the differences between the two are relatively small as well. Considering all of the observations made for Figure 1, we conclude that  has the most dominant effect, followed by *D*(*t*). The role of *F*(*t*) is relatively minor compared to the others.

**Figure 2:**
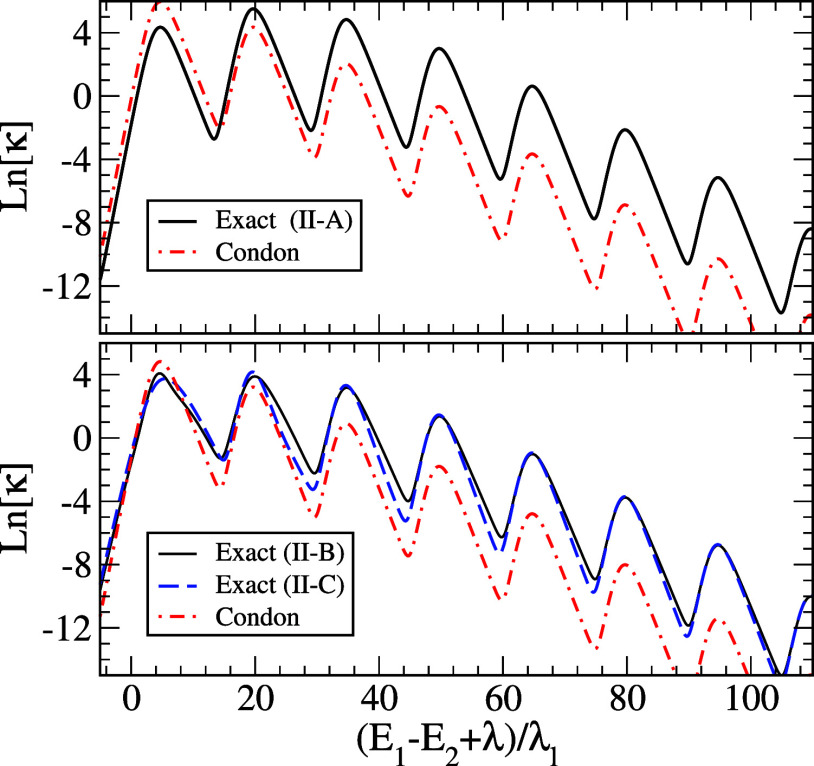
Natural logarithms of scaled rates  (in the units where *k*_B_ = ℏ = ω_c_ = 1), for case II-A (upper panel) and cases II-B and II-C (lower panel), are compared with reference results of a Condon approximation, eq 44, for the same cases.

Figure 2 shows results for cases II-A, B, and C of Table 2, for which the frequency of the vibrational mode is 3 times larger than those for Figure 1. Although the reorganization energy of the single vibrational frequency in this case is smaller than that for Figure 1, a more pronounced vibrational progression effect can be seen. Otherwise, qualitative results are similar to those in Figure 1.

## Application to Azulene

**Figure 3:**
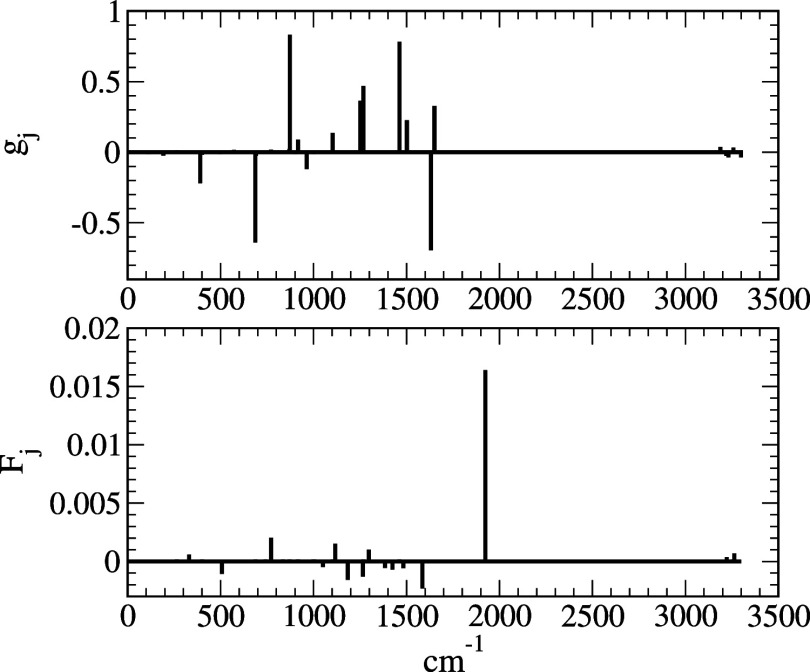
Histograms of *g*_*j*_, which is dimensionless, and *F̃*_*j*_ (in atomic units) versus the wavenumber of normal modes for azulene.

We conducted calculations for an actual molecule, azulene, employing the time-dependent density functional method with M06-2X functional^62^ and 6-31+G* basis. We optimized the molecule in its first excited state in the gas phase and calculated Huang–Rhys (HR) factors and the first NDC terms corresponding to the S_1_ → S_0_ transition for all of the vibrational modes for an appropriate Eckart frame identified through a well-established numerical procedure.^63^Figure 3 shows histograms of HR factors and NDC terms. It is interesting to note that the contribution of CH stretching modes on both HR and first NDC terms is very small for this transition. It is also interesting to note that the contribution to NDC term is dominated by one vibrational frequency at 1,924 cm^–1^, whereas the HR terms are contributed by a group of vibrational modes with smaller frequencies. Due to very small couplings between the two, it is expected that the contribution of *F*(*t*) to the rate is insignificant.

**Figure 4:**
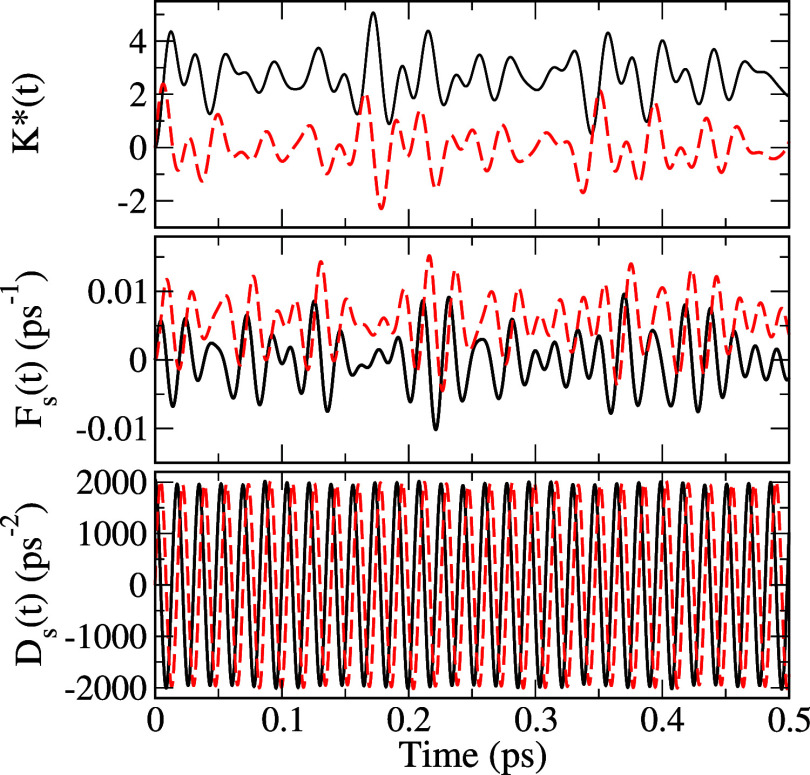
Plots of *(*t*), *F*_s_(*t*) = *F*(*t*)/ℏ(in the unit of ps^–1^), and *D*_s_(*t*) = *D*(*t*)/ℏ^2^ (in the unit of ps^–2^) versus time (in the units of ps) for azulene. Black solid lines are real parts, and red dashed lines are imaginary parts.

Figure 4 shows real and imaginary parts of  (which is dimensionless), and *F*(*t*)/ℏand *D*(*t*)/ℏ^2^ (in the units of ps^–1^ and ps^–2^, respectively) due to all of the vibrational modes determined for an isolated azulene at 300 K. These were calculated directly from eqs 74, 76, and 77. This amounts to calculating eqs 31–33 employing an exact delta function for the three bath spectral densities defined by eqs 27–29. A comparison of the relative magnitudes of *F*(*t*) and *D*(*t*) in Figure 4 clearly shows that the latter can practically account for all contributions of NDC terms for this molecule.

For the calculation of rates, we also included the effects of environments in two different ways. One was to add the contribution of the Ohmic bath, the first term in eq 34, only to , which amounts to adding additional  and  given by eqs 37 and 38 with λ_h_ = 0. This represents the situation where the environmental dynamics can be represented by an Ohmic bath with a spectral range much narrower than the major molecular vibrational frequencies, whereas the modes contributing to derivative couplings remain harmonic.

The other way to include environmental effects was to represent each delta function in the definition of spectral densities, eqs 27–29, with the following normalized Brownian oscillator (BO) model:^64,65^
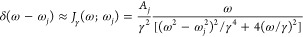
45where *A*_*j*_ is the normalization constant determined such that integration of *J*_γ_(ω;ω_*j*_) over [0,∞] is unity and γ is a parameter representing the friction of the environment. An explicit expression for *A*_*j*_ is provided by eq S51 of the SI. The above BO model behaves like a Lorentzian near ω_*j*_ while being free of unphysical limiting behavior^66,67^ of the latter that is responsible for numerical ambiguities or artifacts. More specifically, unlike the Lorentzian, the above BO model approaches ω = 0+ linearly and decays as ω^–3^ for large ω. These limiting properties make all three bath correlation functions of the present work well-defined and amenable to straightforward numerical integrations. More details of numerical procedure are provided in the SI. In addition, the above BO model represents an actual physical situation where each vibrational mode is weakly coupled to a broad Ohmic bath and can also be used to some extent to represent the broadening due to the anharmonicity^53^ of the bath degrees of freedom.

**Figure 5:**
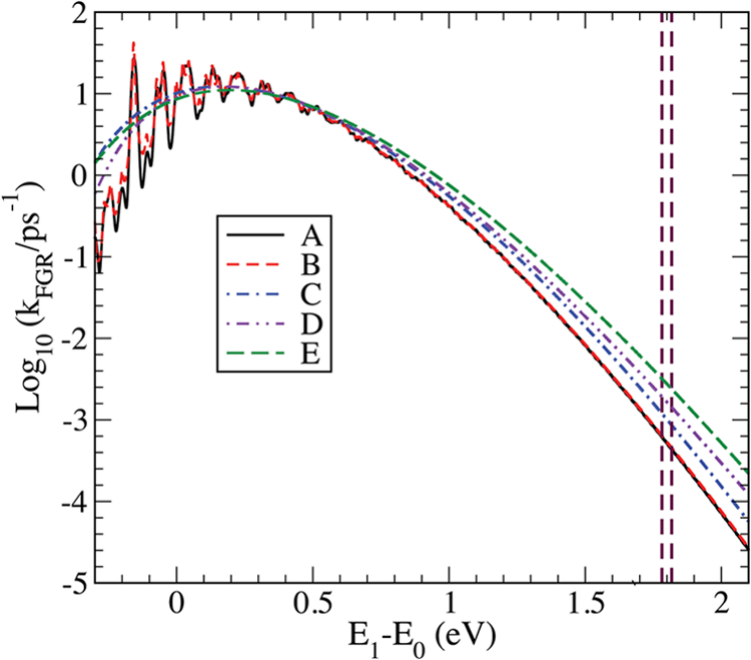
FGR rates of nonradiative decay from S_1_ to S_0_ versus the energy gap *E*_1_–*E*_0_ of azulene for five different bath model parameters provided in Table 3. The two vertical lines represent the minimum and maximum values of the energy gap in the solution.

**Figure 6:**
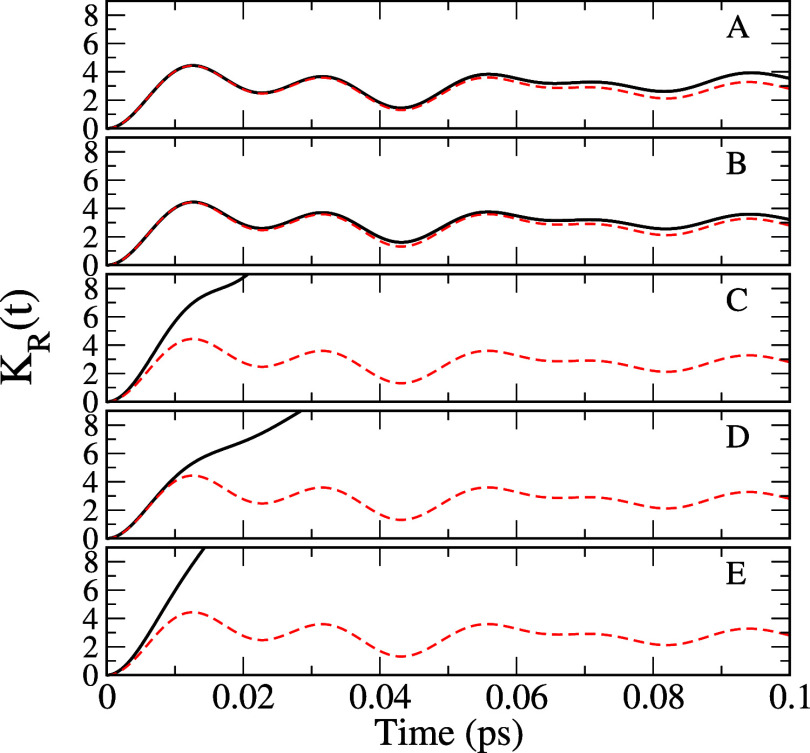
Plots of  versus time for the five different cases A–E of Table 3 for azulene are shown as black lines. The data without bath (which was provided in Figure 4) are shown as red dashed lines as a reference.

Figure 5 shows rates calculated according to eq 26 at 300 K for five different choices as listed in Table 3, where the values of parameters, η, ν̃_c_ = ω_c_/(2*π c*), and γ̃ = γ/(2*π c*) are shown. Figure 6 provides s for the five cases calculated during the short initial time, in comparison with that in the absence of a bath (The same data for a longer time are shown in the top panel of Figure 4.) Similar figures for all other correlation functions are listed in the SI. Two vertical dashed lines in Figure 5 represent the lower and upper bounds of experimental energy gaps^68^ determined in different solution phases at room temperature and correspond, respectively, to 1.7816 and 1.8176 eV. Table 3 also lists the minimum and maximum values of FGR rates at the two values of the energy gap.

**Table 3:** Table of Bath Model Parameters, η and ν̃_c_ = ω_c_/(2*π c*) for the Ohmic Bath Spectral Density Defined by the First Term of eq 34 and Table 1, and γ̃ = γ/(2*π c*), where γ is Defined by eq 45^a^

bath	η	ν̃_c_(cm^–1^)	γ̃ (cm^–1^)	*k*_min_ (ns^–1^)	*k*_max_ (ns^–1^)
A	1	10	0	0.439	0.617
B	0		10	0.453	0.639
C	10	200	0	0.861	1.20
D	0		400	1.43	1.93
E	10	200	400	2.40	3.20

a Rates calculated at two values of the energy gap are shown. *k*_min_ is for *E*_1_–*E*_0_ = 1.8176 eV and *k*_max_ is for *E*_1_–*E*_0_ = 1.7816 eV.

Bath models A and B correspond to the cases where either the explicit Ohmic bath or the friction coefficient in the BO model is much smaller than the other frequencies. Good agreement between the two, especially in the large energy gap limit, suggests that they are close to intrinsic rates without environmental effects. The similarity of  in the short time limit for these two cases, as shown in Figure 6, confirms this as well. Bath models C–E represent intermediate couplings to environments, for which  starts increasing quickly already around *t* = 0.1 ps, as shown in Figure 6. While model C represents the effects of the low-frequency Ohmic bath, model D represents the significant broadening of each vibrational mode due to couplings to other environmental modes. Model E corresponds to a combination of the two.

It is interesting to note that our highest theoretical estimates are comparable to those of Niu et al.^25^ under a similar assumption. The upward trend in the rate we observe for the larger reorganization energy of the environment suggests that stronger environmental effects on both HR and NDC terms can enhance the rate. Additional contributions of Duschinsky effects^25^ may increase the rate. Anharmonic effects can also enhance the rate significantly.^27^ On the other hand, a very recent theoretical work by Landi et al.^49^ reported new theoretical estimates, 62 and 71 ns^–1^, based on direct quantum dynamics calculation and the generating function approach^19^ within the FGR rate, respectively, for a similar harmonic approximation for all of the vibrational modes. It was also reported^49^ that the net effect of the Duschinsky effect is not significant for the value of the energy gap as opposed to the assessment by Shuai and co-workers.^25^ As yet, although most advanced, the computational data by Landi et al.^49^ seem to require further validation to serve as benchmark data. For example, the convergence of the direct quantum dynamics calculation data must be examined carefully. Further clarification of the effects of the window or apodization function within the generating function also seems necessary. Although detailed and clear theoretical analyses within the generating function approach are available for the simple cases^69−72^ of Lorentzian or Gaussian broadening mechanisms, issues^71^ related to the behavior resulting from small and large frequency limits of the Lorentzian function have not been fully resolved. While Gaussian broadening results in consistent rates,^71^ its physical origin, except for two cases, an ensemble of static disorder and strongly coupled environments, is not clearly understood yet. On the other hand, the rates provided in this section are based on more clear physical assumptions and are free of the effects of any extra window or apodization function.

While the theoretical estimates for the nonradiative rate for S_1_ → S_0_ are relatively large when compared to other organic molecules of similar size, they are still at least 2 orders of magnitude smaller than experimental estimates, which were reported^73,74^ to be about 0.5 ps^–1^ but have since been revised^68,75^ later to be about 1 ps^–1^ or faster. This discrepancy is significant and points to the possibility of other mechanisms. Indeed, there have been theoretical and experimental works^76−80^ suggesting that the major mechanism for fast nonradiative decay of azulene from S_1_ is via conical intersection. While the initial theoretical assessment of the mechanism^76^ was revised later,^79^ additional spectroscopic measurements^77,78,80^ and computational study^80^ are still in strong support of decay through a conical intersection point. However, a direct and quantitative demonstration of this mechanism through advanced dynamics calculations still needs to be made. In particular, the relative slowness of the process indicates that some activation process might be involved, even if the decay occurs through a conical intersection. However, even if this scenario were true, why the rate is independent of the temperature is not well understood. Thus, unambiguous and quantitative clarification of the S_1_ → S_0_ nonradiative decay of azulene remains a major theoretical challenge that still needs to be resolved.

## Conclusions

Starting from a general expression for the molecular Hamiltonian in the adiabatic electronic states and nuclear position states, we considered NDC terms carefully and provided a full formal expression for the FGR rate expression for nonadiabatic transitions between adiabatic states. We then showed that a general expression for the FGR rate, eq 22, can be obtained within a quasi-adiabatic approximation, which employs crude adiabatic electronic states determined at the minimum of the initial adiabatic electronic state. This expression turns out to be equivalent to the general expression (without assuming a promoting mode) being used by Shuai and co-workers.^25^ For the case where all of the nuclear dynamics are modeled as displaced harmonic oscillators, we then derived a closed-form expression for the FGR rate, eq 26, which can be used for the general distribution of vibrational modes and arbitrary couplings between Franck–Condon modes and NDC terms, while still accounting for the non-Condon effect on the rate due to nuclear momenta terms exactly. This expression also clarifies that the nonadiabatic FGR rate requires information on not only full vibrational modes coupled to electronic transitions but also projections of derivative coupling terms along different vibrational modes. All of these can be collectively specified by three different bath spectral densities given by eqs 27–29.

We have conducted model calculations for cases where the bath spectral density consists of a low-frequency Ohmic bath (with an exponential cutoff) and a single high-frequency vibrational mode. Results of calculation for sets of parameters in Table 2 demonstrate nontrivial non-Condon effects due to NDC terms. Overall, there is a consistent enhancement of rate with NDC terms due to higher frequencies, making more significant contributions for a larger energy gap between the donor and acceptor in general.

Application of our theoretical expression to the nonradiative decay rate of azulene clarifies the major factors contributing to the decay of the first excited singlet state via direct nonadiabatic coupling. In particular, it is shown that the broadening of spectral density due to environmental effects or an anharmonic contribution can enhance the rate significantly. As of yet, we estimate that such enhancement is not sufficient to account for fast experimental nonradiative decay rates of azulene. Thus, our calculation serves as indirect support for the decay through conical intersection as a major route for fast picosecond time scale decay observed experimentally. Our rate expression makes it possible to clarify this issue further in combination with more extensive dynamics calculations that allow a more satisfactory determination of bath spectral densities.

Results of the present paper offer new insights into rate processes due to NDC terms such as nonradiative decay of near-infrared and short-wave infrared dye molecules^81,82^ that follow the energy gap law.^61,83^ A recent work^84^ demonstrated the importance of NDC terms projected onto all vibrational frequencies of molecules, but the detailed contribution of non-Condon effects has not been clarified yet. New theoretical expressions and model calculations provided here will help determine such effects quantitatively with input from additional computational data, providing all of the relevant spectral densities.

The major focus of the present work was nonadiabatic transitions that can be described well by an FGR rate equation and to derive a closed-form rate expression for the generic case of displaced harmonic oscillator baths. However, further improvement accounting for Duschinsky effects and anharmonicity is feasible and will be the subject of future efforts. In addition, the validity of the molecular Hamiltonian obtained within the quasi-adiabatic approximation, eqs 16–18 (or with eq 20 instead), is not necessarily limited by the assumption of rate behavior. In this sense, applications of various quantum dynamics methods^16,17,31,46,85−106^ to these Hamiltonians would be interesting and can offer various benchmarks for the rate description. This is feasible with an extension or test of known quantum dynamics methods for the types of coupling terms given by eqs 18 or 20. More general expressions provided in the SI can be used for the cases of multiple adiabatic states. Given that quasi-adiabatic approximation in such cases remains valid, an extension of the current FGR rate for multiple states is feasible, which again can be tested against advanced quantum dynamics calculations. Finally, the general formalism developed for this work also serves as a solid framework to go beyond the quasi-adiabatic approximation.

## Appendix: Calculation of Bath Correlation Functions Involving Momenta

### Preliminary Calculations

Consider the following harmonic oscillator bath Hamiltonian and a bath term linear in position

46

47

For the equilibrium density ρ̂*_b_* = *e*^–β*Ĥ_b_*^/*Tr*{*e*^–β*Ĥ_b_*^}, let us first consider the following well-known time correlation function

48

Then, using 1 = e^–*Ŝ*^e^*Ŝ*^ with *Ŝ* = *g*(*b̂*^†^–*b̂*),

49

where λ = ℏ*ω g*^2^ and

50

The trace in eq 49 can be evaluated as follows:

51

where

52

Therefore,

53

Now, consider the following time correlation function:

54

Following a procedure similar to eq 49, we find that

55

Note that *p̂*(*t*) is proportional to *Ŝ*(*t*) as follows:

56

Therefore, the trace operation in eq 55 can be expressed as

57

Following a procedure similar to obtaining eq 51, we can calculate the trace in the above expression as follows:

58

Therefore,

59

Second, let us consider the following time correlation function:

60

Following a procedure similar to eq 49, we find that

61

Employing eq 56 for *t* = 0, we find that the trace in the last line of the above equation can be expressed as

62

The trace in the above expression can be calculated in a way similar to eq 58 as follows:

63

Therefore,

64

Finally, let us consider the following momentum correlation function:

65

Following a procedure similar to eq 49, we find that

66

In the above expression, the trace can be expressed as

67

Following a procedure similar to eqs 58 and 63, the trace in the above expression can be calculated as follows:

68

Taking partial derivatives of the above expression with respect to α_1_ and α_2_, we find that

69

where *K*_*g*_(*t*) is defined by eq 52. Employing the above expression in eq 67 and then using eq 66, we obtain the following expression:

70

### Evaluation of Equation 25

This section describes how the momentum bath correlation function, eq 25, can be evaluated. For the case where *j* ≠ *j*′, it can be calculated as follows:

71

where eqs 53, 59, or 64 in Appendix A have been used for each relevant vibrational mode, and the reorganization energy λ and bath correlation functions are defined as

72

73

74

For the case where *j* = *j*′, eq 25 can be shown to be

75

where eq 53 or 70 has been used.

Employing the above expressions in eq 25, the FGR rate can be expressed in the form of eq 26 with the following expressions for the additional bath correlation functions:

76

*F**(*t*), which is complex conjugate of *F*(*t*), and

77

## Supporting Information Available

Full derivation of Hamiltonians in the adiabatic basis and nonadiabatic derivative couplings; details of quadratic approximation for the bath Hamiltonian; details of Brownian oscillator model for vibrational peaks and calculation of time correlation functions; plots of  (*t*) versus time for the five different cases A–E of Table III in the main text for azulene; plots of *D*_s,R_(*t*) = *D*_R_(*t*)/ℏ^2^ (in the unit of ps^–2^ versus time for the two different cases B and D of Table III in the main text for azulene; plots of *D*_s,I_ (*t*) = *D*_I_(*t*)/ℏ^2^ (in the unit of ps^–2^) versus time for the two different cases B and D of Table III in the main text for azulene; plots of *F*_s,I_ (*t*) = *F*_I_(*t*)/ℏ (in the unit of ps^–1^) versus time for the two different cases B and D of Table III in the main text for azulene (PDF)

## Footnotes

1 More precisely stated, this implies that there are two adiabatic electronic state surfaces constructed by joining infinite numbers of those for fixed nuclear coordinates.

## References

[ref1] Makhov D. V.; Symonds C.; Fernandez-Alberti S.; Shalashilin D. V. Ab initio quantum direct dynamics simulations of ultrafast photochemistry with multiconfigurational Ehrenfest approach. Chem. Phys. 2017, 493, 200–218. 10.1016/j.chemphys.2017.04.003.

[ref2] Tao H. L.; Levine B. G.; Martinez T. J. Ab initio multiple spawning dynamics using mult-state second-order perturbation theory. J. Phys. Chem. A 2009, 113, 13656–13662. 10.1021/jp9063565. 19888736

[ref3] Goings J. J.; Lestrange P. L.; Li X. Real-time time-dependent electronic structure theory. WIREs Comput. Mol. Sci. 2017, e1341 10.1002/wcms.1341.

[ref4] Wang L.; Long R.; Prezhdo O. V. Time domain ab initio modeling of photoinduced dynamics at nanoscale interfaces. Annu. Rev. Phys. Chem. 2015, 66, 549–579. 10.1146/annurev-physchem-040214-121359. 25622188

[ref5] Curchod B. F. E.; Martinez T. J. Ab Initio nonadiabatic quantum molecular dynamics. Chem. Rev. 2018, 118, 3305–3336. 10.1021/acs.chemrev.7b00423. 29465231

[ref6] Guo H.; Worth G.; Domcke W. Quantum dynamics with ab initio potentials. J. Chem. Phys. 2021, 155, 080401 10.1063/5.0066234. 34470339

[ref7] Zhao L.; Tao Z.; Pavosevic F.; Wildman A.; Hammes-Schiffer S.; Li X. Real-time time-dependent nuclear-electronic orbital approach: Dynamics beyond the Born-Oppenheimer approximation. J. Phys. Chem. Lett. 2020, 11, 4052–4058. 10.1021/acs.jpclett.0c00701. 32251589

[ref8] Song H.; Fischer S. A.; Zhang Y.; Cramer C. J.; Mukamel S.; Govind N.; Tretiak S. First principles nonadiabatic excited-state molecular dynamics in NWChem. J. Chem. Theory Comput. 2020, 16, 6418–6427. 10.1021/acs.jctc.0c00295. 32808780

[ref9] Hegger R.; Binder R.; Burghardt I. First-principles quantum and quantum-classical simulations of exciton diffusion in semiconducting polymer chains at finite temperature. J. Chem. Theory Comput. 2020, 16, 5441–5455. 10.1021/acs.jctc.0c00351. 32786907

[ref10] Gao J.; Rossky P. J. The age of direct chemical dynamics. Acc. Chem. Res. 2022, 55, 471–472. 10.1021/acs.accounts.2c00021. 35164506 PMC12967001

[ref11] Deumens E.; Diz A.; Longo R.; Öhrn Y. Time-dependent theoretical treatments of the dynamics of electrons and nuclei in molecular systems. Rev. Mod. Phys. 1994, 66, 917 10.1103/RevModPhys.66.917.

[ref12] Abedi A.; Maitra N. T.; Gross E. K. U. Exact factorization of the time-dependent electron-nuclear wave function. Phys. Rev. Lett. 2010, 105, 123002 10.1103/PhysRevLett.105.123002. 20867633

[ref13] Abedi A.; Maitra N. T.; Gross E. K. U. Correlated electron-nuclear dynamics: Exact factorization of the molecular wavefunction. J. Chem. Phys. 2012, 137, 22A530 10.1063/1.4745836. 23249067

[ref14] Min S. K.; Agostini F.; Tavernelli I.; Gross E. K. U. Ab Initio nonadiabatic dynamics with coupled trajectories: A rigorous approach to quantum (de)coherence. J. Phys. Chem. Lett. 2017, 8, 3048–3055. 10.1021/acs.jpclett.7b01249. 28618782

[ref15] He X.; Wu B.; Shang Y.; Li B.; Cheng X.; Liu J. New phase space formulations and quantum dynamics approaches. WIREs Comput. Mol. Sci. 2022, 12, e1619 10.1002/wcms.1619.

[ref16] Wu B.; He X.; Liu J. Nonadiabatic field on quantum phase space: A century after Ehrenfest. J. Phys. Chem. Lett. 2024, 15, 644–658. 10.1021/acs.jpclett.3c03385. 38205956

[ref17] He X.; Cheng X.; Wu B.; Liu J. Nonadiabatic field with triangle window functions on quantum phase space. J. Phys. Chem. Lett. 2024, 15, 5452–5466. 10.1021/acs.jpclett.4c00793. 38747729 PMC11129318

[ref18] Kubo R. Thermal ionization of trapped electrons. Phys. Rev. 1952, 86, 929 10.1103/PhysRev.86.929.

[ref19] Kubo R.; Toyozawa Y. Application of the method of generating function to radiative and non-radiative transitions of a trapped electron in a crystal. Prog. Theor. Phys. 1955, 13, 160–182. 10.1143/PTP.13.160.

[ref20] Lin S. H. Rate of interconversion of electronic and vibrational energy. J. Chem. Phys. 1966, 44, 3759–3767. 10.1063/1.1726531.

[ref21] Lin S. H. Radiationless transitions in isolated molecules. J. Chem. Phys. 1973, 58, 5760–5768. 10.1063/1.1679200.

[ref22] Nitzan A.; Jortner J. Intramolecular nonradiative transitions in the Non-Condon scheme. J. Chem. Phys. 1972, 56, 3360–3373. 10.1063/1.1677705.

[ref23] Fujimura Y.; Kono H.; Nakajima T. Theory of nonradiative decays in the non-Condon scheme. J. Chem. Phys. 1977, 66, 199–206. 10.1063/1.433654.

[ref24] Mebel A. M.; Hayashi M.; Liang K. K.; Lin S. H. Ab Initio calculations of vibronic spectra and dynamics for small polyatomic molecules: Role of Duschinsky effect. J. Phys. Chem. A 1999, 103, 10674–10690. 10.1021/jp992429m.

[ref25] Niu Y.; Peng Q.; Deng C.; Gao X.; Shuai Z. Theory of excited state decays and optical spectra: Application to polyatomic molecules. J. Phys. Chem. A 2010, 114, 7817–7831. 10.1021/jp101568f. 20666533

[ref26] Peng Q.; Niu Y.; Deng C.; Shuai Z. Vibration correlation function formalism of radiative and non-radiative rates for complex molecules. Chem. Phys. 2010, 370, 215–222. 10.1016/j.chemphys.2010.03.004.

[ref27] Wang Y.; Ren J.; Shuai Z. Evaluating the anharmonicity contributions to the molecular excited internal conversion rates with finite temperature TD-DMRG. J. Chem. Phys. 2021, 154, 214109 10.1063/5.0052804. 34240969

[ref28] Borrelli R.; Peluso A. Perturbative calculation of Franck-Condon integrals: New hints for a rational implementation. J. Chem. Phys. 2008, 129, 064116 10.1063/1.2967183. 18715060

[ref29] Sharf B.; Silbey R. Near resonance interactions in the B-O scheme. The vibronic mechanism pertinent to radiationless transitions and inhomogeneous line broadening. Chem. Phys. Lett. 1971, 9, 125–128. 10.1016/0009-2614(71)80203-8.

[ref30] Orlandi G.; Siebrand W. Vibronic coupling and line broadening in polyatomic molecules. Chem. Phys. Lett. 1971, 8, 473–476. 10.1016/0009-2614(71)80070-2.

[ref31] Tully J. C. Perspective: Nonadiabatic dynamics theory. J. Chem. Phys. 2012, 137, 22A301 10.1063/1.4757762. 23249037

[ref32] Jasper A. W.; Nangia S.; Zhu C.; Truhlar D. G. Non-Born-Oppenheimer molecular dynamics. Acc. Chem. Res. 2006, 39, 101–108. 10.1021/ar040206v. 16489729

[ref33] Izmaylov A. F.; Mendive-Tapia D.; Bearpark M. J.; Robb M. A.; Tully J. C.; Frisch M. J. Nonequilibrium Fermi golden rule for electronic transitions through conical intersection. J. Chem. Phys. 2011, 135, 234106 10.1063/1.3667203. 22191863

[ref34] Kapral R. Progress in the theory of mixed quantum-classical dynamics. Annu. Rev. Chem. Phys. 2006, 57, 129–157. 10.1146/annurev.physchem.57.032905.104702. 16599807

[ref35] Subotnik J. E.; Jain A.; Landry B.; Petit A.; Ouyang W.; Bellonzi N. Understanding the surface hopping view of electronic transitions and decoherence. Annu. Rev. Phys. Chem. 2016, 67, 387–417. 10.1146/annurev-physchem-040215-112245. 27215818

[ref36] Esch M. P.; Levine B. G. An accurate, non-empirical method for incorporating decoherence into Ehrenfest dynamics. J. Chem. Phys. 2021, 155, 214101 10.1063/5.0070686. 34879667

[ref37] Huang D. M.; Green A. T.; Martens C. C. A first principles derivation of energy-conserving momentum jumps in surface hopping simulations. J. Chem. Phys. 2023, 159, 214108 10.1063/5.0178534. 38047505

[ref38] Zhu X.; Yarkony D. R. Quasi-diabatic representations of adiabatic potential energy surfaces coupled by conical intersections including bond breaking: A more general construction procedure and an analysis of the diabatic representation. J. Chem. Phys. 2005, 1, 22A511 10.1063/1.4734315. 23249048

[ref39] Joubert-Doriol L.; Izmaylov A. J. Nonadiabatic Quantum Dynamics with Frozen-Width Gaussians. J. Phys. Chem. A 2018, 122, 6031–6042. 10.1021/acs.jpca.8b03404. 29781620

[ref40] Zhou W.; Mandal A.; Huo P. Quasi-diabatic scheme for nonadiabatic on-the-fly simulations. J. Phys. Chem. Lett. 2019, 10, 7062–7070. 10.1021/acs.jpclett.9b02747. 31665889

[ref41] Prezhdo O. V. Modeling non-adiabatic dynamics in nanoscale and condensed matter systems. Acc. Chem. Res. 2021, 54, 4329–4249. 10.1021/acs.accounts.1c00525. 34756013

[ref42] Shu Y.; Zhang L.; Chen X.; Sun S.; Huang Y.; Truhlar D. G. Nonadiabatic dynamics algorithms with only potential energies and gradients: Curvature-driven coherent switching with decay of mixing and curvature-driven trajectory surface hopping. J. Chem. Theory Comput. 2022, 18, 1320–1328. 10.1021/acs.jctc.1c01080. 35104136

[ref43] Knox R. S. Theory of excitons; Academic Press: New York, 1963.

[ref44] Kenkre V. M.; Reineker P. Exciton Dynamics in Molecular Crystals and Aggregates; Springer: Berlin, 1982.

[ref45] May V.; Kühn O. Charge and Energy Transfer Dynamics in Molecular Systems; Wiley-VCH: Weinheim, Germany, 2011.

[ref46] Jang S. J. Dynamics of Molecular Excitons (Nanophotonics Series); Elsevier: Amsterdam, 2020.

[ref47] Niu Y.; Peng Q.; Shuai Z. Promoting-mode free formalism for excited state radiationless decay process with Duschinsky rotation effect. Sci. China, Ser. B: Chem. 2008, 51, 1153–1158. 10.1007/s11426-008-0130-4.

[ref48] Jang S. Nonadiabatic quantum Liouville equation and master equations in the adiabatic basis. J. Chem. Phys. 2012, 137, 22A536 10.1063/1.4748142. 23249073

[ref49] Landi A.; Landi A.; Leo A.; Peluso A. The rates of non-adiabatic processes in large molecular systems: Toward an effective full-dimensional quantum mechanical approach. J. Chem. Phys. 2024, 160, 174114 10.1063/5.0200345. 38748022

[ref50] Fermi E. Nuclear Physics; University of Chicago Press: Chicago, 1950.

[ref51] Jang S. J.; Rhee Y. M. Modified Fermi’s golden rule rate expressions. J. Chem. Phys. 2023, 159, 014101 10.1063/5.0152804. 37403843

[ref52] Jang S. J.; Mennucci B. Delocalized excitons in natural light harvesting complexes. Rev. Mod. Phys. 2018, 90, 035003 10.1103/RevModPhys.90.035003.

[ref53] Cho K. H.; Jang S. J.; Rhee Y. M. Dynamic embedding of effective harmonic normal mode vibrations in all-atomistic energy gap fluctuations: case study of light harvesting 2 complex. J. Chem. Phys. 2024, 160, 014101 10.1063/5.0206944. 38716847

[ref54] Weiss U. Series in Modern Condensed Matter Physics Vol. 2: Quantum Dissipative Systems; World Scientific: Singapore, 1993.

[ref55] Nitzan A. Chemical Dynamics in Condensed Phases; Oxford University Press: Oxford, 2006.

[ref56] Jang S.; Cao J.; Silbey R. J. On the temperature dependence of molecular line shapes due to linearly coupled phonon bands. J. Phys. Chem. B 2002, 106, 8313–8317. 10.1021/jp0208440.

[ref57] Ritschel G.; Eisfeld A. Analytical representations of bath correlation functions for ohmic and superohmic spectral densities using simple poles. J. Chem. Phys. 2014, 141, 094101 10.1063/1.4893931. 25194358

[ref58] Zaccone A.; Baggioli M. Universal law for the vibrational density of states of liquids. Proc. Natl. Acad. Sci. U.S.A. 2021, 118, e2022303118 10.1073/pnas.2022303118. 33495319 PMC7865170

[ref59] Onuchic J. N.; Beratan D. N.; Hopfield J. J. Some aspects of electron-transfer dynamics. J. Phys. Chem. A 1986, 90, 3707–3721. 10.1021/j100407a045.

[ref60] Sun X.; Geva E. Exact vs. asymptotic spectral densities in the Garg-Onuchic-Ambegaokar charge transfer model and its effect on Fermi’s golden rule rate constants. J. Chem. Phys. 2016, 144, 044106 10.1063/1.4940308. 26827201

[ref61] Jang S. J. A simple generalization of the energy gap law for nonradiative processes. J. Chem. Phys. 2021, 155, 164106 10.1063/5.0068868. 34717346

[ref62] Zhao Y.; Truhlar D. G. The M06 suite of density functionals for main group thermochemistry, thermochemical kinetics, noncovalent interactions, excited states, and transition elements: two new functionals and systematic testing of four M06-class functionals and 12 other functionals. Theor. Chem. Acc. 2008, 120, 215–241. 10.1007/s00214-007-0310-x.

[ref63] Rhee Y. M. Construction of an accurate potential energy surface by interpolation with Cartesian weighting coordinates. J. Chem. Phys. 2000, 113, 6021–6024. 10.1063/1.1315348.

[ref64] Garg A.; Onuchic J. N.; Ambegaokar V. Effect of friction on electron transfer in biomolecules. J. Chem. Phys. 1985, 83, 4491–4503. 10.1063/1.449017.

[ref65] Mukamel S. Principles of Nonlinear Spectroscopy; Oxford University Press: New York, 1995.

[ref66] Sluis K. M.; Gislason E. A. Decay of a quantum-mechanical state described by a truncated Lorentzian energy distribution. Phys. Rev. A 1991, 43, 4581 10.1103/PhysRevA.43.4581. 9905572

[ref67] Elyutin P. V. Natural line shape. JETP Lett. 2008, 88, 496–500. 10.1134/S002136400820006X.

[ref68] Wagner B. D.; Szymanski M.; Steer R. P. Subpicosecond pump-probe measurements of the electronic relaxation rates of the S_1_ states of azulene and related compounds in polar and nonpolar solvents. J. Chem. Phys. 1993, 98, 301–307. 10.1063/1.464675.

[ref69] Nitzan A.; Jortner J. Electronic relaxation of small molecules in a dense medium. Theoret. Chim. Acta 1973, 29, 97–116. 10.1007/BF00529434.

[ref70] Nitzan A.; Jortner J. Nonradiative transition probabilities in the statistical limit. Theoret. Chim. Acta 1973, 30, 217–229. 10.1007/BF00527613.

[ref71] Kühn L. H.; Metz F. Theory of intramolecular radiationless transitions. Chem. Phys. 1978, 33, 137–150. 10.1016/0301-0104(78)87079-7.

[ref72] Freed K. F. Radiationless transitions in molecules. Acc. Chem. Res. 1978, 11, 74–80. 10.1021/ar50122a004.

[ref73] Ippen E. P.; Shank C. V.; Woerner R. L. Picosecond dynamics of azulene. Chem. Phys. Lett. 1977, 46, 20–23. 10.1016/0009-2614(77)85155-5.

[ref74] Shank C. V.; Ippen E. P.; Teschke O.; Fork R. L. Radiationless relaxation processes in azulene. Chem. Phys. Lett. 1978, 57, 433–434. 10.1016/0009-2614(78)85542-0.

[ref75] Schwarzer D.; Troe J.; Schroeder J. S_1_ lifetime of azulene in solution. Ber. Bunsenges. Phys. Chem. 1991, 95, 933–934. 10.1002/bbpc.19910950814.

[ref76] Bearpark M. J.; Bernardi F.; Clifford S.; Olivucci M.; Robb M.; Smith B. R.; Vreven T. The azulene S_1_ state decays via a conical intersection: A CASSCF study with MMVB dynamics. J. Am. Chem. Soc. 1996, 118, 169–175. 10.1021/ja9514555.

[ref77] Diau E. W. G.; Feyter S. D.; Zewail A. H. Direct observation of the femtosecond nonradiative dynamics of azulene in a molecular beam: The anomalous behavior in the isolated molecule. J. Chem. Phys. 1999, 110, 9785–9788. 10.1063/1.478031.

[ref78] Wurzer A. J.; Wilhelm T.; Piel J.; Riedle E. Comprehensive measurement of the S_1_ azulene relaxation dynamics and observation of vibrational wavepacket motion. Chem. Phys. Lett. 1999, 299, 296–302. 10.1016/S0009-2614(98)01288-3.

[ref79] Amatatsu Y.; Komura Y. Reaction coordinate analysis of the S_1_ → S_0_ internal conversion of azulene. J. Chem. Phys. 2006, 125, 174311 10.1063/1.2364891. 17100443

[ref80] Dunlop D.; Ludvíková L.; Banerjee A.; Ottosson H.; Slanina T. Excited-state (anti)aromaticity explains why azulene disobeys Kasha’s rule. J. Am. Chem. Soc. 2023, 145, 21569–21575. 10.1021/jacs.3c07625. 37704031 PMC10557139

[ref81] Friedman H. C.; Cosco E. D.; Atallah T. L.; Jia S.; Sletten E. M.; Caram J. R. Establishing design principles for emissive organic SWIR chromophores from energy gap laws. Chem 2021, 7, P3359–3376. 10.1016/j.chempr.2021.09.001. PMC8664240 34901520

[ref82] Erker C.; Basche T. The energy gap law at work: Emission yield and rate fluctuations of single NIR emitters. J. Am. Chem. Soc. 2022, 144, 14053–14056. 10.1021/jacs.2c07188. 35904975

[ref83] Englman R.; Jortner J. The energy gap law for radationless transitions in large molecules. Mol. Phys. 1970, 18, 145–164. 10.1080/00268977000100171.

[ref84] Ramos P.; Friedman H.; Li B. Y.; Garcia C.; Sletten E.; Caram J. R.; Jang S. J. Nonadiabatic derivative couplings through multiple Franck-Condon modes dictate the energy gap law for near and short-wave infrared dye molecules. J. Phys. Chem. Lett. 2024, 15, 1802–1810. 10.1021/acs.jpclett.3c02629. 38329913

[ref85] Liang R.; Cotton S. J.; Binder R.; Hegger R.; Burghardt I.; Miller W. H. The symmetrical quasi-classical approach to electronically nonadiabatic dynamics applied to ultrafast exciton migration processes in semiconducting polymers. J. Chem. Phys. 2018, 149, 044101 10.1063/1.5037815. 30068189

[ref86] Makri N. Time-dependent quantum methods for large systems. Annu. Rev. Phys. Chem. 1999, 50, 167–191. 10.1146/annurev.physchem.50.1.167. 15012410

[ref87] Huo P.; Coker D. F. Consistent schemes for non-adiabatic dynamics derived from partial linearized density matrix propagation. J. Chem. Phys. 2012, 137, 22A535 10.1063/1.4748316. 23249072

[ref88] Makri N. Iterative blip-summed path integral for quantum dynamics in strongly dissipative environments. J. Chem. Phys. 2017, 146, 134101 10.1063/1.4979197. 28390349

[ref89] Kelly A.; Markland T. E. Efficient and accurate surface hopping for long time nonadiabatic quantum dynamics. J. Chem. Phys. 2013, 139, 014104 10.1063/1.4812355. 23822290

[ref90] Mandal A.; Yamijala S. S. R. K. C.; Huo P. F. Quasi-diabatic representation for nonadiabatic dynamics propagation. J. Chem. Phys. 2018, 14, 1828–1840. 10.1021/acs.jctc.7b01178. 29489359

[ref91] Beck M. H.; Jackie A.; Worth G. A.; Meyer H. D. The multiconfiguration time-dependent Hartree (MCTDH) method: a highly efficient algorithm for propagating wavepackets. Phys. Rep. 2000, 324, 1–105. 10.1016/S0370-1573(99)00047-2.

[ref92] Manthe U. A multilayer multiconfigurational time-dependent Hartree approach for quantum dynamics on general potential energy surfaces. J. Chem. Phys. 2008, 128, 164116 10.1063/1.2902982. 18447430

[ref93] Burghardt I.; Giri K.; Worth G. A. Multimode quantum dynamics using Gaussian wavepackets: The Gaussian-based multiconfiguration time-dependent Hartree (G-MCTDH) method applied to the absorption spectrum of pyrazine. J. Chem. Phys. 2008, 129, 174104 10.1063/1.2996349. 19045330

[ref94] Wang H.; Thoss M. A multilayer multiconfiguration time-dependent Hartree simulation of the reaction coordinate spin-boson model employing an interaction picture. J. Chem. Phys. 2017, 146, 124112 10.1063/1.4978901. 28388113

[ref95] Breuer H. P.; Kappler B.; Petruccione F. The time-convolutionless projection operator technique in the quantum theory of dissipation and decoherence. Ann. Phys. 2001, 291, 36–70. 10.1006/aphy.2001.6152.

[ref96] Shi Q.; Geva E. A new approach to calculating the memory kernel of the generalized quantum master equation for an arbitrary system-bath coupling. J. Chem. Phys. 2003, 119, 12063–12076. 10.1063/1.1624830. 15268091

[ref97] Shi Q.; Geva E. A semiclassical generalized quantum master equation for an arbitrary system-bath coupling. J. Chem. Phys. 2004, 120, 10647–10658. 10.1063/1.1738109. 15268091

[ref98] Ishizaki A.; Fleming G. R. Unified treatment of quantum coherent and incoherent hopping dynamics in electronic energy transfer: Reduced hierarchy equation approach. J. Chem. Phys. 2009, 130, 234111 10.1063/1.3155372. 19548715

[ref99] Tanimura Y. Stochastic Liouville, Langevin, Fokker-Planck, and Master Equation approaches to quantum dissipative systems. J. Phys. Soc. Jpn. 2006, 75, 082001 10.1143/JPSJ.75.082001.

[ref100] Tanimura Y. Real-time and imaginary-time quantum hierarchical Fokker-Planck equation. J. Chem. Phys. 2015, 142, 144110 10.1063/1.4916647. 25877565

[ref101] Zheng X.; Xu R. X.; Xu J.; Jin J. S.; Hu J.; Yan Y. J. Hierarchical equations of motion for quantum dissipation and quantum transport. Prog. Chem. 2012, 24, 1129–1152.

[ref102] de Vega I.; Alonso D. Dynamics of non-Markovian open quantum systems. Rev. Mod. Phys. 2017, 89, 015001 10.1103/RevModPhys.89.015001.

[ref103] Lyu N.; Mulvihill E.; Soley M. B.; Geva E.; Batista V. S. Tensor-train thermo-field memory kernels for generalized quantum master equations. J. Chem. Theory Comput. 2023, 19, 1111–1129. 10.1021/acs.jctc.2c00892. 36719350

[ref104] Hsieh C.; Liu J.; Duan C.; Cao J. A nonequilibrium variational polaron theory to study quantum heat transport. J. Phys. Chem. C 2019, 123, 17196–17204. 10.1021/acs.jpcc.9b05607.

[ref105] Yang L.; Devi M.; Jang S. Polaronic quantum master equation theory of inelastic and coherent resonance energy transfer for soft systems. J. Chem. Phys. 2012, 137, 024101 10.1063/1.4732309. 22803522

[ref106] Jang S. J. Partially polaron-transformed quantum master equation for exciton and charge transport dynamics. J. Chem. Phys. 2022, 157, 104107 10.1063/5.0106546. 36109233

