# Supplementary material for: Fermi’s Golden Rule Rate Expression for Transitions Due to Nonadiabatic Derivative Couplings in the Adiabatic Basis
Source: J Chem Theory Comput. 2025 Feb 13;21(4):1850–64. doi: 10.1021/acs.jctc.4c00590 (PMC11866763; doi:10.1021/acs.jctc.4c00590)
Supplement: Supplementary file 1 — ct4c00590_si_001.pdf [file ct4c00590_si_001.pdf]

Supporting Information: Fermi's Golden Rule Rate Expression for Transitions Due to Nonadiabatic Derivative Couplings in the Adiabatic Basis

Seogjoo J. Jang,[1, a] Byeong Ki Min,[2] and Young Min Rhee[2]
[1] *Department of Chemistry and Biochemistry, Queens College, City University of New York, 65-30 Kissena Boulevard, Queens, New York 11367, USA & PhD Programs in Chemistry and Physics, Graduate Center of the City University of New York, New York 10016, USA*
[2] *Department of Chemistry, Korea Advanced Institute of Science and Technology, Daejeon 34141, Korea*

(Dated: 31 January 2025)

I. HAMILTONIANS IN THE ADIABATIC BASIS AND NONADIABATIC DERIVATIVE COUPLINGS

A. Isolated molecules

1. Hamiltonian

Consider a molecular Hamiltonian with N_e electrons and N_u nuclei. For now, let us assume[1] that there are no other degrees of freedom involved. The corresponding Hamiltonian in atomic units can be expressed as[2]

$$\hat{H} = \hat{H}_{en} + \hat{H}_{nu}, \tag{S1}$$

where

$$\hat{H}_{en} = \sum_{\mu=1}^{N_e} \frac{\hat{\mathbf{p}}_\mu^2}{2} - \sum_{\mu=1}^{N_e}\sum_{c=1}^{N_u} \frac{Z_c e^2}{|\hat{\mathbf{r}}_\mu - \hat{\mathbf{R}}_c|^2} + \frac{1}{2}\sum_{\mu=1}^{N_e}\sum_{\nu\neq\mu} \frac{1}{|\hat{\mathbf{r}}_\mu - \hat{\mathbf{r}}_\nu|}, \tag{S2}$$

$$\hat{H}_{nu} = \sum_{c=1}^{N_u} \frac{\hat{\mathbf{P}}_c}{2M_c} + \frac{1}{2}\sum_{c=1}^{N_u}\sum_{c'\neq c} \frac{Z_c Z_{c'}}{|\hat{\mathbf{R}}_c - \hat{\mathbf{R}}_{c'}|}. \tag{S3}$$

In the above expressions, $\hat{\mathbf{p}}_\mu$ and $\hat{\mathbf{r}}_\mu$ are momentum and position operators of an electron labeled with μ, and $\hat{\mathbf{P}}_c$, $\hat{\mathbf{R}}_c$, M_c, and Z_c are momentum operator, position operator, mass, and charge of a nucleus labeled with c.

Let us consider the adiabatic electronic Hamiltonian $\hat{H}_{en}(\mathbf{R})$, which is the same as Eq. (S2) except that the nuclear position operator vector $\hat{\mathbf{R}} \equiv (\hat{\mathbf{R}}_1, \cdots, \hat{\mathbf{R}}_{N_u})^T$ in Eq. (S2) is replaced with corresponding vector parameter: $\mathbf{R} \equiv (\mathbf{R}_1, \cdots, \mathbf{R}_{N_u})^T$. Then, one can define the following adiabatic electronic states and eigenvalues:

$$\hat{H}_{en}(\mathbf{R})|\psi_{e,k}(\mathbf{R})\rangle = E_{e,k}(\mathbf{R})|\psi_{e,k}(\mathbf{R})\rangle, \tag{S4}$$

where k collectively denotes the set of all quantum numbers that are necessary to completely specify the adiabatic electronic states defined for the given nuclear coordinate $\mathbf{R}$. Thus, given that all such states can be identified, the following completeness relation holds in the electronic space:

$$\hat{1}_{e,\mathbf{R}} = \sum_k |\psi_{e,k}(\mathbf{R})\rangle\langle\psi_{e,k}(\mathbf{R})|. \tag{S5}$$

In the above expression, the subscript $\mathbf{R}$ denotes that the resolution is with respect to adiabatic electronic states defined at $\mathbf{R}$. This subscript is used to make it clear that each component of the resolution is dependent on $\mathbf{R}$ even though in principle the electronic identity operator itself is independent of nuclear coordinates. In practice, due to the approximations made for the adiabatic electronic states and the truncation of the summation, the expansion is not complete. Thus, in practice, the righthand side of Eq. (S5) ends up being an approximation and becomes dependent upon $\mathbf{R}$.

At a formal level, it is always possible to assume the existence of Eq. (S5). When this identity resolution is used carefully, it is possible[3] to decompose the molecular Hamiltonian $\hat{H}$ into adiabatic and nonadiabatic components such that both are expressed in terms of outer products (in Dirac notation) involving adiabatic electronic states and nuclear position states in a consistent manner. Thus, the molecular Hamiltonian, Eq. (S1), can be expressed as[4]

$$\hat{H} = \hat{H}_{ad} + \frac{1}{2}\sum_{\alpha=1}^{3N_u}\left(\hat{P}_\alpha \hat{F}_\alpha + \hat{F}_\alpha \hat{P}_\alpha\right) + \hat{S}, \tag{S6}$$

where α denotes each one dimensional Cartesian component and $\hat{H}_{ad}$ is the adiabatic approximation for the molecular Hamiltonian given by

$$\hat{H}_{ad} = \int d\mathbf{R} \sum_k |\mathbf{R}\rangle|\psi_{e,k}(\mathbf{R})\rangle\Big\{ - \sum_{\alpha=1}^{3N_u} \frac{\hbar^2}{2M_\alpha}\frac{\partial^2}{\partial R_\alpha^2} + U_k(\mathbf{R})\Big\}\langle\psi_{e,k}(\mathbf{R})|\langle\mathbf{R}|, \tag{S7}$$

with

$$U_k(\mathbf{R}) = E_{e,k}(\mathbf{R}) + \frac{1}{2}\sum_{c=1}^{N_u}\sum_{c'\neq c} \frac{Z_c Z_{c'}}{|\mathbf{R}_c - \mathbf{R}_{c'}|}. \tag{S8}$$

In Eq. (S6), $\hat{F}_\alpha$ represents the first nonadiabatic derivative coupling (NDC) term,

$$\hat{F}_\alpha = \int d\mathbf{R} \sum_k \sum_{k'} |\mathbf{R}\rangle|\psi_{e,k}(\mathbf{R})\rangle F_{\alpha,kk'}(\mathbf{R})\langle\psi_{e,k'}(\mathbf{R})|\langle\mathbf{R}|, \tag{S9}$$

where

$$F_{\alpha,kk'}(\mathbf{R}) = \frac{\hbar}{iM_\alpha}\langle\psi_{e,k}(\mathbf{R})|\left(\frac{\partial}{\partial R_\alpha}|\psi_{e,k'}(\mathbf{R})\rangle\right). \tag{S10}$$

[a] Electronic mail: seogjoo.jang@qc.cuny.edu

Note that $\hat{P}_\alpha \hat{F}_\alpha$ in Eq. (S6) contains part of the conventional second NDC term. The last term in Eq. (S6) represents the remaining second NDC term,

$$\hat{S} = \int d\mathbf{R} \sum_k \sum_{k'} |\mathbf{R}\rangle |\psi_{e,k}(\mathbf{R})\rangle S_{kk'}(\mathbf{R}) \langle \psi_{e,k'}(\mathbf{R})| \langle \mathbf{R}|, \tag{S11}$$

where each component involves products of first NDC terms[3] as follows:

$$S_{kk'}(\mathbf{R}) = \frac{1}{2} \sum_{\alpha=1}^{3N_u} \sum_{k''} M_\alpha F_{\alpha,kk''}(\mathbf{R}) F_{\alpha,k''k'}(\mathbf{R}). \tag{S12}$$

Thus, Eq. (S6) with above clarification shows that all NDC terms can be determined once full information on functional form of Eq. (S10) is known. From the condition that $\langle \psi_{e,k}(\mathbf{R}) | \psi_{e,k'}(\mathbf{R}) \rangle = \delta_{kk'}$, it is easy to show[3] that

$$F_{\alpha,kk'}(\mathbf{R}) = F^*_{\alpha,k'k}(\mathbf{R}). \tag{S13}$$

Thus, it is clear that $\hat{F}_\alpha$ defined by Eq. (S9) is Hermitian. This also means that the diagonal component $F_{\alpha,kk}(\mathbf{R})$ is always real. Thus, if all the electronic eigenfunctions $\langle \mathbf{r} | \psi_{e,j}(\mathbf{R}) \rangle$ can be expressed as real-valued functions, $F_{\alpha,kk}(\mathbf{R})$ defined as Eq. (S10) is always zero. On the other hand, in the presence of magnetic fields or for other cases where the adiabatic electronic eigenstate needs to be complex valued, it does not have to be zero.

The expressions given by Eqs. (S6)-(S10) are equivalent to standard expressions, which are typically expressed in terms of those projected onto particular adiabatic electronic and nuclear wave functions. Compared to these expressions, our expressions here provide complete nonadiabatic coupling Hamiltonian terms as standalone Hermitian operators in the most general way, and also makes it possible to understand approximations involved dynamics employing the Hamiltonian.

2. Eigenstates of the adiabatic Hamiltonian and their nonadiabatic couplings

Let us denote the eigenstate of the nuclear degrees of freedom for an adiabatic potential energy surface $U_k(\mathbf{R})$ with eigenvalue E_{n_k} as $|\chi_{n_k}\rangle$. Then,

$$\left(\sum_{\alpha=1}^{3N_u} \frac{\hat{P}_\alpha^2}{2M_\alpha} + U_k(\hat{\mathbf{R}}) \right) |\chi_{n_k}\rangle = E_{n_k} |\chi_{n_k}\rangle, \tag{S14}$$

or equivalently,

$$\left(-\sum_{\alpha=1}^{3N_u} \frac{\hbar^2}{2M_\alpha} \frac{\partial^2}{\partial R_\alpha^2} + U_k(\mathbf{R}) \right) \chi_{n_k}(\mathbf{R}) = E_{n_k} \chi_{n_k}(\mathbf{R}), \tag{S15}$$

where $\chi_{n_k}(\mathbf{R}) = \langle \mathbf{R} | \chi_{n_k} \rangle$. Then, let us introduce the following adiabatic vibronic state defined in the direct product space of electronic and nuclear degrees of freedom:

$$\begin{aligned} |\Psi_{k,n}\rangle &= \int d\mathbf{R} (|\mathbf{R}\rangle \langle \mathbf{R} | \chi_{n_k} \rangle) \otimes |\psi_{e,k}(\mathbf{R})\rangle \\ &= \int d\mathbf{R}\ \chi_{n_k}(\mathbf{R}) |\mathbf{R}\rangle |\psi_{e,k}(\mathbf{R})\rangle, \end{aligned} \tag{S16}$$

where in the second line we have omitted the direct product symbol $\otimes$ between $|\mathbf{R}\rangle$ and $|\psi_{e,k}(\mathbf{R})\rangle$. Then, application of $\hat{H}_{ad,k}$ to $|\Psi_{k,n}\rangle$ results in

$$\begin{aligned} &\hat{H}_{ad,k} |\Psi_{k,n}\rangle \\ &= \int d\mathbf{R} \int d\mathbf{R}' |\mathbf{R}\rangle |\psi_{e,k}(\mathbf{R})\rangle \left\{ -\sum_{\alpha=1}^{3N_u} \frac{\hbar^2}{2M_\alpha} \frac{\partial^2}{\partial R_\alpha^2} + U_k(\mathbf{R}) \right\} \\ &\qquad \times \langle \psi_{e,k}(\mathbf{R}) | \psi_{e,k}(\mathbf{R}') \rangle \langle \mathbf{R} | \mathbf{R}' \rangle \chi_{n_k}(\mathbf{R}') \\ &= \int d\mathbf{R} |\mathbf{R}\rangle |\psi_{e,k}(\mathbf{R})\rangle \left\{ -\sum_{\alpha=1}^{3N_u} \frac{\hbar^2}{2M_\alpha} \frac{\partial^2}{\partial R_\alpha^2} + U_k(\mathbf{R}) \right\} \chi_{n_k}(\mathbf{R}) \\ &= E_{n_k} \int d\mathbf{R} |\mathbf{R}\rangle |\psi_{e,k}(\mathbf{R})\rangle \chi_{n_k}(\mathbf{R}) = E_{n_k} |\Psi_{k,n}\rangle. \end{aligned} \tag{S17}$$

Thus, $|\Psi_{k,n}\rangle$ is an eigenstate of $\hat{H}_{ad,k}$. In a similar manner, it is straightforward to show that $\hat{H}_{ad,k'} |\Psi_{k,n}\rangle = 0$ for $k \neq k'$.

Now let us consider application of nonadiabatic terms as follows.

$$\begin{aligned} \frac{1}{2} \sum_{\alpha=1}^{3N_u} \left(\hat{P}_\alpha \hat{F}_\alpha + \hat{F}_\alpha \hat{P}_\alpha \right) |\Psi_{k,n}\rangle &= \frac{1}{2} \sum_{\alpha=1}^{3N_u} \sum_{k'} \sum_{k''} \int d\mathbf{R} \int d\mathbf{R}' \left\{ \hat{P}_\alpha |\mathbf{R}'\rangle |\psi_{e,k'}(\mathbf{R}')\rangle F_{\alpha,k'k''}(\mathbf{R}') \langle \psi_{e,k''}(\mathbf{R}')| \langle \mathbf{R}'| \right. \\ &\qquad \left. + |\mathbf{R}'\rangle |\psi_{e,k'}(\mathbf{R}')\rangle F_{\alpha,k'k''}(\mathbf{R}') \langle \psi_{e,k''}(\mathbf{R}')| \langle \mathbf{R}'| \hat{P}_\alpha \right\} |\psi_{e,k}(\mathbf{R})\rangle |\mathbf{R}\rangle \langle \mathbf{R} | \chi_{n_k} \rangle \\ &= \frac{1}{2} \sum_{\alpha=1}^{3N_u} \left\{ \sum_{k'} \int d\mathbf{R} \hat{P}_\alpha |\mathbf{R}\rangle |\psi_{e,k'}(\mathbf{R})\rangle F_{\alpha,k'k}(\mathbf{R}) \langle \mathbf{R} | \chi_{n_k} \rangle \right. \\ &\qquad \left. + \sum_{k'} \sum_{k''} \int d\mathbf{R} \int d\mathbf{R}' |\mathbf{R}'\rangle |\psi_{e,k'}(\mathbf{R}')\rangle F_{\alpha,k'k''}(\mathbf{R}') \langle \psi_{e,k''}(\mathbf{R}') | \psi_{e,k}(\mathbf{R}) \rangle \langle \mathbf{R}' | \hat{P}_\alpha | \mathbf{R} \rangle \langle \mathbf{R} | \chi_{n_k} \rangle \right\}. \end{aligned} \tag{S18}$$

Taking inner product of the above equation with $\langle\Psi_{l,m}| = \int d\mathbf{R}''\ \langle\psi_{e,l}(\mathbf{R}'')|\langle\mathbf{R}''|\chi^*_{l_m}(\mathbf{R}'') = \int d\mathbf{R}''\ \langle\psi_{e,l}(\mathbf{R}'')|\langle\chi_{l_m}|\mathbf{R}''\rangle\langle\mathbf{R}''|$ on the right hand side, we obtain

$$\langle\Psi_{l,m}|\frac{1}{2}\sum_{\alpha=1}^{3N_u}\left(\hat{P}_\alpha\hat{F}_\alpha+\hat{F}_\alpha\hat{P}_\alpha\right)|\Psi_{k,n}\rangle=\frac{1}{2}\sum_{\alpha=1}^{3N_u}\left\{\sum_{k'}\int d\mathbf{R}\int d\mathbf{R}''\langle\chi_{l_m}|\mathbf{R}''\rangle\langle\mathbf{R}''|\hat{P}_\alpha|\mathbf{R}\rangle\langle\psi_{e,l}(\mathbf{R}'')|\psi_{e,k'}(\mathbf{R})\rangle F_{\alpha,k'k}(\mathbf{R})\langle\mathbf{R}|\chi_{n_k}\rangle\right.$$
$$\left.+\sum_{k''}\int d\mathbf{R}\int d\mathbf{R}''\langle\chi_{l_m}|\mathbf{R}''\rangle F_{\alpha,lk''}(\mathbf{R}'')\langle\psi_{e,k''}(\mathbf{R}'')|\psi_{e,k}(\mathbf{R})\rangle\langle\mathbf{R}''|\hat{P}_\alpha|\mathbf{R}\rangle\langle\mathbf{R}|\chi_{n_k}\rangle\right\}. \tag{S19}$$

Noting that $\langle\mathbf{R}''|\hat{P}_\alpha|\mathbf{R}\rangle=(\hbar/i)(\partial/\partial R''_\alpha)\delta(\mathbf{R}''-\mathbf{R})=-(\hbar/i)(\partial/\partial R_\alpha)\delta(\mathbf{R}''-\mathbf{R})$ and then conducting integration by part, we obtain

$$\begin{aligned}
&\langle\Psi_{l,m}|\frac{1}{2}\sum_{\alpha=1}^{3N_u}\left(\hat{P}_\alpha\hat{F}_\alpha+\hat{F}_\alpha\hat{P}_\alpha\right)|\Psi_{k,n}\rangle\\
&=\frac{\hbar}{2i}\sum_{\alpha=1}^{3N_u}\left\{\sum_{k'}\int d\mathbf{R}\int d\mathbf{R}''\langle\chi_{l_m}|\mathbf{R}''\rangle\delta(\mathbf{R}''-\mathbf{R})\frac{\partial}{\partial R_\alpha}\langle\psi_{e,l}(\mathbf{R}'')|\psi_{e,k'}(\mathbf{R})\rangle F_{\alpha,k'k}(\mathbf{R})\langle\mathbf{R}|\chi_{n_k}\rangle\right.\\
&\qquad\left.+\sum_{k''}\int d\mathbf{R}\int d\mathbf{R}''\langle\chi_{l_m}|\mathbf{R}''\rangle F_{\alpha,lk''}(\mathbf{R}'')\delta(\mathbf{R}''-\mathbf{R})\frac{\partial}{\partial R_\alpha}\langle\psi_{e,k''}(\mathbf{R}'')|\psi_{e,k}(\mathbf{R})\rangle\langle\mathbf{R}|\chi_{n_k}\rangle\right\}\\
&=\frac{\hbar}{2i}\sum_{\alpha=1}^{3N_u}\left\{\int d\mathbf{R}\langle\chi_{l_m}|\mathbf{R}\rangle\left(\sum_{k'}\frac{iM_\alpha}{\hbar}F_{\alpha,lk'}(\mathbf{R})F_{\alpha,k'k}(\mathbf{R})\langle\mathbf{R}|\chi_{n_k}\rangle+\frac{\partial}{\partial R_\alpha}F_{\alpha,lk}(\mathbf{R})\langle\mathbf{R}|\chi_{n_k}\rangle\right)\right.\\
&\qquad\left.+\int d\mathbf{R}\langle\chi_{l_m}|\mathbf{R}\rangle\left(\sum_{k''}F_{\alpha,lk''}(\mathbf{R})\frac{iM_\alpha}{\hbar}F_{\alpha,k''k}(\mathbf{R})\langle\mathbf{R}|\chi_{n_k}\rangle+F_{\alpha,lk}(\mathbf{R})\frac{\partial}{\partial R_\alpha}\langle\mathbf{R}|\chi_{n_k}\rangle\right)\right\}\\
&=\sum_{\alpha=1}^{3N_u}\left\{\int d\mathbf{R}\chi^*_{l_m}(\mathbf{R})\chi_{n_k}(\mathbf{R})\left(\sum_{k'}M_\alpha F_{\alpha,lk'}(\mathbf{R})F_{\alpha,k'k}(\mathbf{R})+\left(\frac{\hbar}{2i}\frac{\partial}{\partial R_\alpha}F_{\alpha,lk}(\mathbf{R})\right)\right)\right.\\
&\qquad\left.+\int d\mathbf{R}\chi^*_{l_m}(\mathbf{R})F_{\alpha,lk}(\mathbf{R})\frac{\hbar}{i}\frac{\partial}{\partial R_\alpha}\chi_{n_k}(\mathbf{R})\right\}.
\end{aligned} \tag{S20}$$

Note the extra terms containing sum of $F_{\alpha,lk'}(\mathbf{R})F_{\alpha,k'k}(\mathbf{R})$ in the above expression, which appear due to non-orthogonality of adiabatic states. Interestingly, these are the same as the matrix element of the remaining second derivative term $\hat{S}$ as shown below.

$$\langle\Psi_{l,m}|\hat{S}|\Psi_{k,n}\rangle=\frac{1}{2}\sum_{\alpha=1}^{3N_u}\int d\mathbf{R}\chi^*_{l_m}(\mathbf{R})\chi_{n_k}(\mathbf{R})\left(\sum_{k'}M_\alpha F_{\alpha,lk'}(\mathbf{R})F_{\alpha,k'k}(\mathbf{R})\right). \tag{S21}$$

3. Helllman-Feynman expression for Derivative coupling terms

For the case where $k \neq k'$ and for non-degenerate $E_{e,k}(\mathbf{R})$ and $E_{e,k'}(\mathbf{R})$, the Hellmann-Feynman theorem[5,6] can be used to obtain an alternative expression for $F_{\alpha,kk'}(\mathbf{R})$, which is obtained by taking the derivative of the following identity:

$$\langle\psi_{e,k}(\mathbf{R})|\hat{H}_e(\mathbf{R})|\psi_{e,k'}(\mathbf{R})\rangle=E_{e,k}(\mathbf{R})\delta_{kk'}. \tag{S22}$$

Taking derivative of the above identity with respect to R_α, we obtain

$$\begin{aligned}
&\left(\frac{\partial}{\partial R_\alpha}\langle\psi_{e,k}(\mathbf{R})|\right)\hat{H}_e(\mathbf{R})|\psi_{e,k'}(\mathbf{R})\rangle\\
&+\langle\psi_{e,k}(\mathbf{R})|\left(\frac{\partial\hat{H}_e(\mathbf{R})}{\partial R_\alpha}\right)|\psi_{e,k'}(\mathbf{R})\rangle\\
&+\langle\psi_{e,k}(\mathbf{R})|\hat{H}_e(\mathbf{R})\left(\frac{\partial}{\partial R_\alpha}|\psi_{e,k'}(\mathbf{R})\rangle\right)\\
&=\frac{iM_\alpha}{\hbar}F_{\alpha,kk'}(\mathbf{R})(E_{e,k}(\mathbf{R})-E_{e,k'}(\mathbf{R}))\\
&+\langle\psi_{e,k}(\mathbf{R})|\frac{\partial\hat{H}_e(\mathbf{R})}{\partial R_\alpha}|\psi_{e,k'}(\mathbf{R})\rangle=\frac{\partial E_{e,k}(\mathbf{R})}{\partial R_\alpha}\delta_{kk'}.
\end{aligned} \tag{S23}$$

where the fact that $F^*_{\alpha,k'k}(\mathbf{R}) = F_{\alpha,kk'}(\mathbf{R})$ has been used in the second equality. Since we assumed that $k \neq k'$, the righthand side of the above equation is zero. Thus, given that $E_{e,k'}(\mathbf{R}) \neq E_{e,k}(\mathbf{R})$, Eq. (S23) results in the following expression:

$$F_{\alpha,kk'}(\mathbf{R}) = \frac{i\hbar}{M_\alpha} \frac{\langle \psi_{e,k}(\mathbf{R})|(\partial \hat{H}_e(\mathbf{R})/\partial R_\alpha)|\psi_{e,k'}(\mathbf{R})\rangle}{E_{e,k}(\mathbf{R}) - E_{e,k'}(\mathbf{R})}. \tag{S24}$$

The above Hellmann-Feynman expression makes it easy to evaluate off-diagonal derivative coupling between non-degenerate states. Note that the above expression also clarifies that the derivative coupling diverges between two degenerate states unless the numerator also vanishes. Such case of divergence is known as conical intersection, for which a wealth of both theoretical and computational studies are available now. However, in the present work, we only consider cases where $F_{\alpha,kk'}(\mathbf{R})$ remains finite and relatively small. These cases become important for nonradiative decay of near infrared and short wavelength infrared dye molecules, for which nonradiative decay processes exhibit the energy gap law behavior, and for dynamics in the excited state manifold in regions far from conical intersections.

4. Normal mode representation

Let us consider the case where the electronic state is in the adiabatic state $|\psi_1(\mathbf{R})\rangle$, for which $U_1(\mathbf{R})$ is the potential energy function for the nuclear degrees of freedom. We assume that the nuclear coordinate $\mathbf{R}$ is defined in the Eckart frame[7–10] with respect to $\mathbf{R}_1^g$. In principle, this can be identified as follows. First, $\mathbf{R}'$ and the minimum energy structure for state 1, $\mathbf{R}'^g_1$, can be defined in any center-of-mass coordinate frame with respect to which the molecule is static. Then, applying a pseudo-rotation matrix that satisfies the second Eckart condition[8] with respect to $\mathbf{R}'^g_1$, a new coordinate frame can be identified. The nuclear coordinate vectors in this rotated frame are labeled as $\mathbf{R}$ and $\mathbf{R}_1^g$. It is known that identifying the pseudo-rotation matrix satisfying the Eckart condition is nontrivial, but there are well-established practical procedures.[8–10]

We denote the mass-weighted nuclear coordinates as $\tilde{\mathbf{R}}$ such that $\tilde{R}_\alpha = \sqrt{M_\alpha} R_\alpha$. Let us denote the mass-weighted nuclear coordinates that make $U_1(\mathbf{R})$ minimum as $\tilde{\mathbf{R}}_1^g$. Then, expanding $U_1(\mathbf{R})$ around $\tilde{\mathbf{R}}_1^g$ with respect to relative mass-weighted coordinates, $\tilde{\mathbf{R}} - \tilde{\mathbf{R}}_1^g$, up to the second order and diagonalizing the resulting Hessian matrix, one can determine all of normal vibrational modes and frequencies, $q_{1,j}$ and $\omega_{1,j}$ with $j = 1, \cdots, N_{v,1}$, where $N_{v,1}$ is the total number of normal mode vibrations for the vibrational motion around $\tilde{\mathbf{R}}_1^g$ in the electronic state 1. The transformation from Cartesian coordinates to these normal modes are defined as follows:

$$\begin{aligned} q_{1,j} &= \sum_\alpha L_{1,j\alpha}(\tilde{R}_\alpha - \tilde{R}^g_{1,\alpha}) \\ &= \sum_\alpha L_{1,j\alpha}\sqrt{M_\alpha}(R_\alpha - R^g_{1,\alpha}), j = 1, \cdots, N_{v,1}. \end{aligned} \tag{S25}$$

Thus, assuming that an Eckart frame that fully decouples the rotational and vibrational degrees of freedom can be found, the nuclear Hamiltonian operator for the mass-weighted coordinates on the adiabatic electronic state surface of 1 can be expressed as

$$\begin{aligned} \sum_\alpha \frac{\hat{\tilde{P}}_\alpha^2}{2} + U_1(\hat{\mathbf{R}}) &\approx \hat{H}_{1,\mathrm{tr-rot}} + U_1(\mathbf{R}_1^g) \\ &+ \sum_{j=1}^{N_{v,1}} \left(\frac{1}{2}\hat{p}_{1,j}^2 + \frac{\omega_{1,j}^2}{2}\hat{q}_{1,j}^2 \right) + \Delta U_1(\hat{\mathbf{q}}_1), \end{aligned} \tag{S26}$$

where $\hat{p}_{1,j}$ is the canonical momentum operator for $\hat{q}_{1,j}$, $\hat{H}_{1,\mathrm{tr-rot}}$ represents the translation of the center-of-mass and rotational motion around $\mathbf{R}_1^g$, and $\Delta U_1(\hat{\mathbf{q}})$ is the remaining anharmonic term of the potential energy function $U_1(\mathbf{R})$, with arguments expressed in terms of normal coordinates.

Now, let us consider the case where the transition to adiabatic electronic state $|\psi_2(\mathbf{R})\rangle$ occurs, for which the potential energy is $U_2(\mathbf{R})$. This can also be expanded around its minimum energy nuclear coordinates denoted as $\mathbf{R}_2^g$. However, in such expansion, it is important to recognize first that $\mathbf{R}$ is already defined in the Eckart frame with respect to $\mathbf{R}_1^g$, which does not necessarily satisfy the second Eckart condition[8] for $\mathbf{R}_2^g$. This has the following two consequences:

1. It is not guaranteed that the nuclear Hamiltonian defined in the electronic state $|\psi_{e,2}(\mathbf{R})\rangle$ can be decomposed into translation-rotation and vibrational parts as in Eq. (S26).

2. Purely vibrational displacement from $\mathbf{R}_1^g$ may bear some rotational component around $\mathbf{R}_2^g$.

At the moment, complete resolution of the above two issues seems not possible in general. While these issues may be mitigated by adopting curvilinear internal coordinates, whether it results in actual advantage is not clear.[11] Thus, we use Cartesian coordinates and invoke additional assumptions here. First, we assume that the non-uniqueness[8,10] in the choice of the Eckart frame for $\mathbf{R}_1^g$ can be utilized such that $\mathbf{R}_2^g$ is maximally aligned with $\mathbf{R}_1^g$. This will minimize the coupling term between the rotation and vibration parts for the displacement around $\mathbf{R}_2^g$, which we assume to be small enough and can thus be ignored. Similarly, we assume that the projection of pure vibrational components around $\mathbf{R}_1^g$ onto rotational part around $\mathbf{R}_2^g$ can be discarded.

With approximations and assumptions as noted above, which can always be tested for a given molecular system, we can expand $U_2(\mathbf{R})$ with respect to $\tilde{\mathbf{R}} - \tilde{\mathbf{R}}_2^g$ around $\tilde{\mathbf{R}}_2^g$

and identify the normal mode and frequency, $q_{2,j}$ and $\omega_{2,j}$, for $j = 1, \cdots, N_{v,2}$, where $N_{v,2}$ is the total number of normal mode vibrations for the vibrational motion around $\mathbf{R}_2^g$. These are related to mass-weighted cartesian coordinates in the best Eckart frame, as prescribed above, by the following transformation,

$$q_{2,j} = \sum_\alpha L_{2,j\alpha}(\tilde{R}_\alpha - \tilde{R}^g_{2,\alpha}) = \sum_\alpha L_{2,j\alpha}\sqrt{M_\alpha}(R_\alpha - R^g_{2,\alpha}), j = 1, \cdots, N_{v,2}. \tag{S27}$$

Thus, we can make the following approximation:

$$\sum_\alpha \frac{\hat{\tilde{P}}^2_\alpha}{2} + U_2(\hat{\mathbf{R}}) \approx \hat{H}_{2,\mathrm{tr-rot}} + U_2(\mathbf{R}_2^g) + \sum_{j=1}^{N_{v,2}} \left(\frac{1}{2}\hat{p}^2_{2,j} + \frac{\omega^2_{2,j}}{2}\hat{q}^2_{2,j}\right) + \Delta U_2(\hat{\mathbf{q}}_2). \tag{S28}$$

In the above expression, $\hat{H}_{2,\mathrm{tr-rot}}$ represents the translation of the center-of-mass and rotational motion around $\mathbf{R}_2^g$, and $\Delta U_2(\hat{\mathbf{q}}_2)$ is the remaining anharmonic term that is assumed to be expressed fully in terms of normal vibrational modes.

For the case where $N_{v,1} = N_{v,2} = N_v$, $q_{1,j}$ and $q_{2,j}$ can be related by the Duschinsky rotation matrix $\mathbf{J}$ and a displacement vector $\mathbf{K}$ as follows:

$$q_{2,j} = \sum_{k=1}^{N_v} J_{jk}q_{1,k} + K_j \ . \tag{S29}$$

5. Expressions in the subspace of two electronic states in the quasi-adiabatic approximation

On the basis of general expressions provided up to so far, we here provide detailed expressions for approximations for the Hamiltonian in the subspace of two adiabatic electronic states within the quasi-adiabatic approximation. For convenience, we also refer to all the nuclear degrees of freedom as bath. First, the zeroth order Hamiltonian can be expressed as

$$\hat{H}_0 \approx \left\{\hat{H}_{1,b} + U_1(\mathbf{R}_1^g) + S_{11}(\tilde{\mathbf{R}}_1^g)\right\}|\psi_{e,1}\rangle\langle\psi_{e,1}| + \left\{\hat{H}_{2,b} + U_2(\mathbf{R}_1^g) + S_{22}(\tilde{\mathbf{R}}_1^g)\right\}|\psi_{e,2}\rangle\langle\psi_{e,2}|, \tag{S30}$$

where

$$\hat{H}_{1,b} = \hat{H}_{1,\mathrm{tr-rot}} + \sum_{j=1}^{N_{v,1}} \left(\frac{1}{2}\hat{p}^2_{1,j} + \frac{\omega^2_{1,j}}{2}\hat{q}^2_{1,j}\right) + \Delta U_1(\hat{\mathbf{q}}_1), \tag{S31}$$

$$\hat{H}_{2,b} = \hat{H}_{2,\mathrm{tr-rot}} + \sum_{j=1}^{N_{v,2}} \left(\frac{1}{2}\hat{p}^2_{2,j} + \frac{\omega^2_{2,j}}{2}\hat{q}^2_{2,j}\right) + \Delta U_2(\hat{\mathbf{q}}_2) + U_2(\mathbf{R}_2^g) - U_2(\mathbf{R}_1^g). \tag{S32}$$

Similarly, $F_{\alpha,12}(\mathbf{R}_1^g)$ can be expressed as

$$F_{\alpha,12}(\mathbf{R}_1^g) = \frac{i\hbar}{\sqrt{M_\alpha}} \frac{\langle\psi_{e,1}|\left(\partial\hat{H}_e(\mathbf{R})/\partial\tilde{R}_\alpha\right)|_{\tilde{\mathbf{R}}=\tilde{\mathbf{R}}_1^g}|\psi_{e,2}\rangle}{E_{e,1}(\mathbf{R}_1^g) - E_{e,2}(\mathbf{R}_1^g)}, \tag{S33}$$

where note that

$$E_{e,1}(\mathbf{R}_1^g) - E_{e,2}(\mathbf{R}_1^g) = U_1(\mathbf{R}_1^g) - U_2(\mathbf{R}_1^g). \tag{S34}$$

Let us assume that the coupling Hamiltonian $\hat{H}_c$ is independent of translation or rotation in the body fixed frame corresponding to the minimum energy structure for the electronic state 1, which is consistent with neglecting the translation-rotation part of the nuclear Hamiltonian in Eq. (S26). Then, $\hat{H}_c$ can be expressed only in terms of those involving normal vibrational modes for the state 1 as described below. To show this, let us first consider the following matrix element of the NDC terms within the quasi-adiabatic approximation:

$$\langle\mathbf{R}|\sum_\alpha F_{\alpha,12}(\mathbf{R}_1^g)|\psi_{e,1}\rangle\langle\psi_{e,2}|\hat{P}_\alpha|\Psi\rangle = \sum_\alpha F_{\alpha,12}(\mathbf{R}_1^g)|\psi_{e,1}\rangle\langle\psi_{e,2}|\langle\mathbf{R}|\hat{P}_\alpha|\Psi\rangle = \sum_\alpha F_{\alpha,12}(\mathbf{R}_1^g)|\psi_{e,1}\rangle\langle\psi_{e,2}|\frac{\hbar}{i}\frac{\partial}{\partial R_\alpha}\langle\mathbf{R}|\Psi\rangle, \tag{S35}$$

where $|\Psi\rangle$ is an arbitrary state in the total Hilbert space including both electronic and nuclear degrees of freedom. Given that all the translation and rotational degrees of freedom are frozen,

$$\frac{\partial}{\partial R_\alpha} = \sum_{j=1}^{N_{v,1}} \frac{\partial q_{1,j}}{\partial R_\alpha}\frac{\partial}{\partial q_{1,j}} = \sum_{j=1}^{N_{v,1}} L_{1,j\alpha}\sqrt{M_\alpha}\frac{\partial}{\partial q_{1,j}}. \tag{S36}$$

Therefore,

$$\frac{\hbar}{i}\frac{\partial}{\partial R_\alpha}\langle\mathbf{R}|\Psi\rangle = \sum_{j=1}^{N_{v,1}} L_{1,j\alpha}\sqrt{M_\alpha}\frac{\hbar}{i}\frac{\partial}{\partial q_{1,j}}\langle\mathbf{R}|\Psi\rangle = \sum_{j=1}^{N_{v,1}} L_{1,j\alpha}\sqrt{M_\alpha}\langle\mathbf{R}|\hat{p}_{1,j}|\Psi\rangle. \tag{S37}$$

Similarly, $F_{\alpha,12}(\mathbf{R}_1^g)$ can be expressed in terms of $\tilde{F}_{j,12}$ as follows:

$$F_{\alpha,12}(\mathbf{R}_1^g) = \frac{1}{\sqrt{M_\alpha}}\sum_{j'=1}^{N_{v,1}} L_{1,\alpha j'}\tilde{F}_{j',12}. \tag{S38}$$

As a result, we find that

$$\langle \mathbf{R}| \sum_\alpha F_{\alpha,12}(\mathbf{R}_1^g)|\psi_{e,1}\rangle\langle\psi_{e,2}|\hat{P}_\alpha|\Psi\rangle$$
$$= \langle \mathbf{R}| \sum_\alpha \sum_{j=1}^{N_{v,1}} \sum_{j'=1}^{N_{v,2}} L_{1,j\alpha}L_{1,j'\alpha}|\psi_{e,1}\rangle\langle\psi_{e,2}|\hat{p}_{1,j}\tilde{F}_{j',12}|\Psi\rangle$$
$$= \langle \mathbf{R}| \sum_{j=1}^{N_{v,1}} \sum_{j'=1}^{N_{v,2}} |\psi_{e,1}\rangle\langle\psi_{e,2}|\hat{p}_{1,j}\tilde{F}_{j',12}|\Psi\rangle, \quad \text{(S39)}$$

where the fact that $\sum_\alpha L_{1,j\alpha}L_{1,j'\alpha} = \delta_{jj'}$ has been used. The above identity holds for an arbitrary vector $\mathbf{R}$, which does not have any translation and rotational degree, and for any state $|\Psi\rangle$. Therefore, the above identity amounts to the following general identity:

$$\sum_\alpha F_{\alpha,12}(\mathbf{R}_1^g)|\psi_{e,1}\rangle\langle\psi_{e,2}|\hat{P}_\alpha$$
$$= \sum_{j=1}^{N_{v,1}} \sum_{j'=1}^{N_{v,2}} |\psi_{e,1}\rangle\langle\psi_{e,2}|\hat{p}_{1,j}\tilde{F}_{j',12}. \quad \text{(S40)}$$

Combining this with its Hermitian conjugate, we obtain the following expression for $\hat{H}_c$:

$$\hat{H}_c = \sum_{j=1}^{N_{v,1}} \hat{p}_{1,j}\left\{\tilde{F}_{j,12}|\psi_{e,1}\rangle\langle\psi_{e,2}| + \tilde{F}^*_{j,12}|\psi_{e,2}\rangle\langle\psi_{e,1}|\right\}, \quad \text{(S41)}$$

where

$$\tilde{F}_{j,12} = i\hbar\frac{\langle\psi_{e,1}|\left(\partial\hat{H}_e(\mathbf{R})/\partial q_{1,j}|_{\mathbf{R}=\mathbf{R}_1^g}\right)|\psi_{e,2}\rangle}{E_{e,1}(\mathbf{R}_1^g) - E_{e,2}(\mathbf{R}_1^g)}. \quad \text{(S42)}$$

B. Molecules in liquid or solid environments

For molecules in liquid or solid environments, translation, rotational, and vibrational modes of molecules are in general coupled to those of environmental degrees of freedom. On the other hand, since the total number of degrees of freedom for the system plus environment is virtually infinite, we can ignore its translation and rotational motion. Thus, all degrees of freedom can be viewed as vibrational. Let us denote the position vector for the environmental degrees of freedom collectively as $\mathbf{X}$. Then, following a procedure similar to obtaining the Hamiltonian for isolated molecules, one can obtain following expressions for the zeroth order Hamiltonian term:

$$\hat{H}_0 \approx \Big\{\sum_{\alpha=1}^{3N_u}\frac{\hat{P}_\alpha^2}{2M_\alpha} + \sum_\xi \frac{\hat{P}_\xi^2}{2m_\xi}$$
$$+U_1(\hat{\mathbf{R}},\hat{\mathbf{X}}) + S_{11}(\mathbf{R}_1^g,\mathbf{X}_1^g)\Big\}|\psi_{e,1}\rangle\langle\psi_{e,1}|$$
$$+\Big\{\sum_{\alpha=1}^{3N_u}\frac{\hat{P}_\alpha^2}{2M_\alpha} + \sum_\xi \frac{\hat{P}_\xi^2}{2m_\xi}$$
$$+U_2(\hat{\mathbf{R}},\hat{\mathbf{X}}) + S_{22}(\mathbf{R}_1^g,\mathbf{X}_1^g)\Big\}|\psi_{e,2}\rangle\langle\psi_{e,2}| \quad \text{(S43)}$$

where $\hat{P}_\xi$ is the momentum conjugate to the ξ component of $\mathbf{X}$ and m_ξ is its mass. On the other hand, the NDC terms can still be assumed to depend only on the molecular vibrational degrees of freedom directly as follows:

$$\hat{H}_c \approx \sum_{\alpha=1}^{3N_u}\hat{P}_\alpha\left(F_{\alpha,12}(\mathbf{R}_1^g,\mathbf{X}_1^g)|\psi_{e,1}\rangle\langle\psi_{e,2}|\right.$$
$$\left.+F^*_{\alpha,12}(\mathbf{R}_1^g,\mathbf{X}_1^g)|\psi_{e,2}\rangle\langle\psi_{e,1}|\right). \quad \text{(S44)}$$

Making expansion of $U_k(\hat{\mathbf{R}},\hat{\mathbf{X}})$ up to the second order of displacements around $\mathbf{R}_k^g$ and $\mathbf{X}_k^g$, and determining normal modes in the extended space of molecular and environmental degrees of freedom, $\hat{H}_0$ and $\hat{H}_c$ can still be expressed in terms of these normal coordinates. Thus, the resulting formal expression for $\hat{H}_0$ and $\hat{H}_c$ remain the same except that detailed forms of $\Delta U_k(\mathbf{q}_k)$s are different and each normal mode in this case is a linear combination of molecular and environmental degrees of freedom.

II. QUADRATIC APPROXIMATION FOR BATH HAMILTONIANS

For the case where contributions of anharmonic terms are small or negligible, quadratic approximations can be made given that subtle assumptions concerning Eckart frame are well justified. The resulting expressions can be combined into the following expressions for the zeroth order Hamiltonian:

$$\hat{H}_0 \approx \left\{E_1^0 + \hat{H}_{b,1}\right\}|\psi_{e,1}\rangle\langle\psi_{e,1}|$$
$$+\left\{E_2^0 + \hat{H}_{b,2}\right\}|\psi_{e,2}\rangle\langle\psi_{e,2}|, \quad \text{(S45)}$$

where

$$E_1^0 = U_1(\mathbf{R}_1^g) + S_{11}(\mathbf{R}_1^g), \quad \text{(S46)}$$
$$E_2^0 = U_2(\mathbf{R}_1^g) + S_{22}(\mathbf{R}_1^g), \quad \text{(S47)}$$

and

$$\hat{H}_{b,1} = \sum_{j=1}^{N_{v,1}}\left(\frac{1}{2}\hat{p}_{1,j}^2 + \frac{\omega_{1,j}^2}{2}\hat{q}_{1,j}^2\right), \quad \text{(S48)}$$

$$\hat{H}_{b,2} = \sum_{j=1}^{N_{v,2}}\left(\frac{1}{2}\hat{p}_{2,j}^2 + \frac{\omega_{2,j}^2}{2}\hat{q}_{2,j}^2\right)$$
$$+U_2(\mathbf{R}_2^g) - U_2(\mathbf{R}_1^g). \quad \text{(S49)}$$

III. BROWNIAN OSCILLATOR SPECTRAL DENSITY TO REPRESENT VIBRATIONAL PEAKS

The actual vibrational peaks become broadened due to relaxation to other modes, anharmonicity, and couplings to environments. Given that all of these sources of broadening can be modeled as an Ohmic bath with spectral range larger than the given vibrational frequency, the corresponding vibrational spectral density can be modeled by the following Brownian oscillator bath spectral density:[12]

$$J_{\rm BO}(\omega;\omega_j,\gamma)=\frac{A_j}{\gamma^2}\frac{\omega}{\left[(\omega^2-\omega_j^2)^2/\gamma^4+4(\omega/\gamma)^2\right]}, \quad \text{(S50)}$$

where γ is the friction coefficient of the environment[13] and A_j can be determined so that the integration of the above spectral density over $[0,\infty]$ to be normalized as follows:

$$A_j=\begin{cases} 4\sqrt{R_j}\left[\frac{\pi}{2}+\tan^{-1}\left(\frac{(R_j-1)}{2\sqrt{R_j}}\right)\right]^{-1} & , \ R_j>0 \\ 2 & , \ R_j=0 \\ 8\sqrt{-R_j}\left[\ln\left(\frac{1-R_j+2\sqrt{-R_j}}{1-R_j-2\sqrt{-R_j}}\right)\right]^{-1} & , \ R_j<0 \end{cases}, \quad \text{(S51)}$$

with $R_j=(\omega_j/\gamma)^2-1$. The above spectral density behaves almost like a Lorentzian near $\omega=\omega_j$ while approaching $\omega=0$ linearly and decays fast enough (as $1/\omega^3$) for large value of ω. Thus, we use this model to account for the broadening of delta function peaks in the bath spectral densities. For the case of $\mathcal{J}(\omega)$, the resulting expression is

$$\begin{aligned}\mathcal{J}(\omega)&=\pi\hbar\sum_j\frac{A_j}{\gamma^2}\frac{\omega\omega_j^2g_j^2}{\left[(\omega^2-\omega_j^2)^2/\gamma^4+4(\omega/\gamma)^2\right]}\\ &=2\pi^2c\hbar\sum_j\frac{A_j}{\tilde\gamma^2}\frac{\tilde\nu\tilde\nu_j^2g_j^2}{\left[(\tilde\nu^2-\tilde\nu_j^2)^2/\tilde\gamma^4+4(\tilde\nu/\tilde\gamma)^2\right]},\end{aligned} \quad \text{(S52)}$$

where in the second line $\tilde\nu=\omega/(2\pi c)$, $\tilde\nu_j=\omega_j/(2\pi c)$, and $\tilde\gamma=\gamma/(2\pi c)$. Although integration for the calculation of $\mathcal{K}(t)$ employing the above spectral density is well defined, care should be taken in actual numerical integration due to the fact that $\mathcal{J}(\omega)/\omega^2$ becomes singular at $\omega=0$ and because of highly oscillatory nature of the integrand in the large ω limit. It is also important to note that the above definition of the spectral density results in small changes in the reorganization energy, which we take into consideration in the calculation of rates.

To handle the numerical issues noted above, we divide $\mathcal{J}(\omega)$ into two components such that

$$\mathcal{J}(\omega)=\mathcal{J}_0(\omega)+\delta\mathcal{J}(\omega), \quad \text{(S53)}$$

where $\mathcal{J}_0(\omega)$ is a combination of Ohmic bath spectral densities given by

$$\mathcal{J}_0(\omega)=2A\omega e^{-\omega/\Omega_c}\left(1-\frac{1}{2}e^{-\omega/\Omega_c}\right), \quad \text{(S54)}$$

with

$$A=\pi\hbar\sum_j\frac{\gamma^2g_j^2}{\omega_j^3}A_j. \quad \text{(S55)}$$

Since $\mathcal{J}_0(\omega)$ is the difference of two Ohmic baths with exponential cutoff, the closed form expressions for the real and imaginary parts of $\mathcal{K}(t)$ provided in the main text, Eqs. (37) and (38) with $\lambda_h=0$, can be used for each of the two terms, resulting in the following expressions:

$$\begin{aligned}\mathcal{K}_{0,R}(t)\approx&\frac{2A}{\pi\hbar}\left\{\frac{1}{2}\ln(1+\tau_{1,0}^2)+\ln(1+\tau_{1,1}^2)+\ln(1+\tau_{1,2}^2)\right.\\ &\left.+\frac{2(1+5\theta_1/2)}{\theta_1}\left[\tau_{1,5/2}\tan^{-1}(\tau_{1,5/2})-\frac{1}{2}\ln(1+\tau_{1,5/2}^2)\right]\right\}\\ &-\frac{A}{2\pi\hbar}\left\{\frac{1}{2}\ln(1+\tau_{2,0}^2)+\ln(1+\tau_{2,1}^2)+\ln(1+\tau_{2,2}^2)\right.\\ &\left.+\frac{2(1+5\theta_1/2)}{\theta_2}\left[\tau_{2,5/2}\tan^{-1}(\tau_{2,5/2})-\frac{1}{2}\ln(1+\tau_{2,5/2}^2)\right]\right\},\end{aligned} \quad \text{(S56)}$$

$$\mathcal{K}_{0,I}(t)=\frac{2A}{\pi\hbar}\tan^{-1}(\tau_{1,0})-\frac{A}{2\pi\hbar}\tan^{-1}(\tau_{1,0}), \quad \text{(S57)}$$

with $\theta_1=\beta\hbar\Omega_c$, $\tau_{1,n}=\Omega_ct/(1+n\theta_1)$, $\theta_2=\beta\hbar\Omega_c/2$, and $\tau_{2,n}=(\Omega_c/2)t/(1+n\theta_2)$. All the expressions provided above are valid for any choice of Ω_c. For the present work, we chose $\Omega_c=\gamma$.

The leading order for the second term in Eq. (S53), $\delta\mathcal{J}(\omega)$, is of ω^3 as follows:

$$\begin{aligned}\delta\mathcal{J}(\omega)&=\mathcal{J}(\omega)-\mathcal{J}_0(\omega)\\ &=\pi\hbar\sum_jA_j\frac{\gamma^2g_j^2}{\omega_j^2}\left\{\frac{1}{\omega_j^2}\left(1-\frac{2\gamma^2}{\omega_j^2}\right)+\frac{1}{\omega_c^2}\right\}\omega^3+\cdots\end{aligned} \quad \text{(S58)}$$

Therefore, numerical integration involving $\delta\mathcal{J}(\omega)$ in the limit of $\omega\to0+$ can be done in a simple manner using a finite interval. As yet, care should still be taken for the limit of $\omega\to\infty$ because of strong oscillatory nature of the integrand while $\delta J(\omega)/\omega^2$ decays proportional to $1/\omega$ in the limit of $\omega\to\infty$. We handle this issue by linearizing only the non-oscillatory part of the integrand and then performing exact integration for each interval. To explain this more clearly, let us consider the following integral:

$$F_c(t;\omega_1,\omega_2)=\int_{\omega_1}^{\omega_2}d\omega f(\omega)(1-\cos(\omega t)), \quad \text{(S59)}$$

where $f(\omega)$ is a smooth function that remains finite during the interval. Then, linearizing $f(\omega)$ within the interval of $[\omega_1,\omega_2]$, the above integral can be calculated explicitly. For $t\neq0$, the resulting expression is

$$\begin{aligned}F_c(t;\omega_1,\omega_2)\approx&(\omega_2-\omega_1)\frac{(f_2+f_1)}{2}\\ &-\frac{1}{t}\left(f_2\sin(\omega_2t)-f_1\sin(\omega_1t)\right)\\ &-\frac{\cos(\omega_2t)-\cos(\omega_1t)}{t^2}\frac{(f_2-f_1)}{(\omega_2-\omega_1)},\end{aligned} \quad \text{(S60)}$$

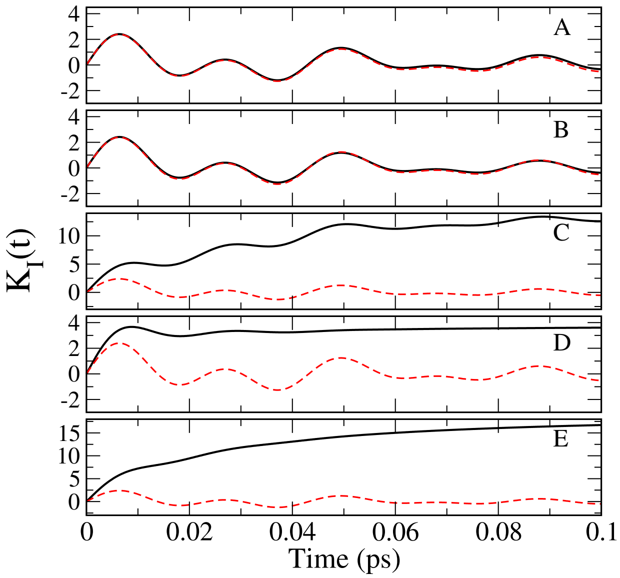

FIG. S1. Plots of $\mathcal{K}_I(t)$ versus time for the five different cases A-E of Table 3 in the main text for azulene, shown as black lines. The data without bath are provided as red dashed lines for reference.

where $f_1 = f(\omega_1)$, $f_2 = f(\omega_2)$. For $t = 0$, $F_c(0; \omega_1, \omega_2) = 0$. Note that the error resulting from this approximation is of order $O((\omega_2 - \omega_1)^3)$. In a similar manner, we find that, for $t \neq 0$,

$$F_s(t; \omega_1, \omega_2) = \int_{\omega_1}^{\omega_2} d\omega f(\omega) \sin(\omega t) \approx \frac{1}{t} \left(\cos(\omega_1 t) f_1 - \cos(\omega_2 t) f_2\right) + \frac{(f_2 - f_1)}{t^2} \frac{(\sin(\omega_2 t) - \sin(\omega_1 t))}{\omega_2 - \omega_1}. \quad (S61)$$

The above expression approaches the proper limit for $t = 0$, $F_s(0; \omega_1, \omega_2) = 0$, and also has errors of order $O((\omega_2 - \omega_1)^3)$ as well.

In summary, the real and imaginary parts of the time correlation function due to $\delta\mathcal{J}(\omega)$ can be calculated as follows:

$$\delta\mathcal{K}_R(t) \approx \sum_{n=0}^{M} F_c(t; n\delta\omega, (n+1)\delta\omega), \quad (S62)$$

$$\delta\mathcal{K}_I(t) \approx \sum_{n=0}^{M} F_s(t; n\delta\omega, (n+1)\delta\omega), \quad (S63)$$

where $F_c(t; n\delta\omega, (n+1)\delta\omega)$ is given by Eq. (S60) with the following definition:

$$f(\omega) = \frac{1}{\pi\hbar} \frac{\delta\mathcal{J}(\omega)}{\omega^2} \coth\left(\frac{\beta\hbar\omega}{2}\right). \quad (S64)$$

and $F_s(t; n\delta\omega, (n+1)\delta\omega)$ is given by Eq. (S61) with the following definition:

$$f(\omega) = \frac{1}{\pi\hbar} \frac{\delta\mathcal{J}(\omega)}{\omega^2}. \quad (S65)$$

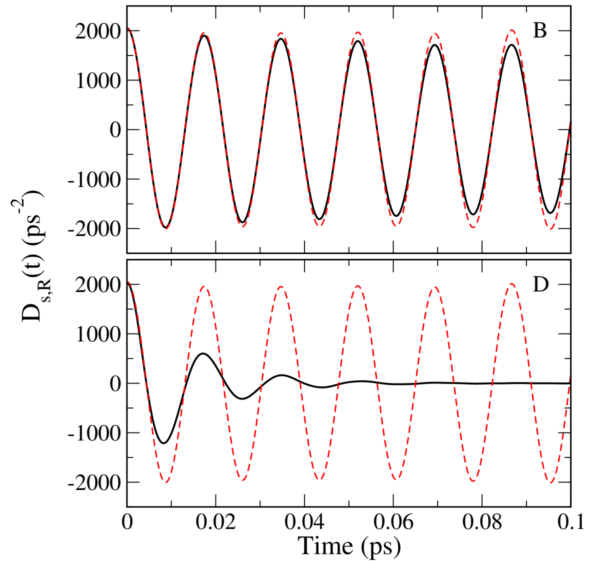

FIG. S2. Plots of $D_{s,R}(t) = D_R(t)/\hbar^2$ (in the unit of ps^{-2} versus time for the two different cases B and D of Table 3 in the main text for azulene, shown as black lines. The data without bath are provided as red dashed lines for reference.

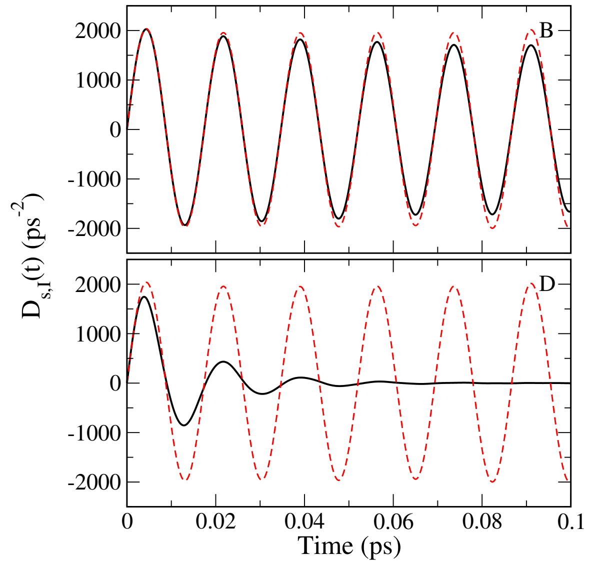

FIG. S3. Plots of $D_{s,I}(t) = D_I(t)/\hbar^2$ (in the unit of ps^{-2}) versus time for the two different cases B and D of Table 3 in the main text for azulene, shown as black lines. The data without bath are provided as red dashed lines for reference.

We confirmed numerically that the approximations, Eqs. (S62) and (S63), converge for sufficiently small $\delta\omega$ and large M. For the calculations provided in the main text, we chose $\delta\tilde{\nu} = \delta\omega/(2\pi c) = 0.1$ cm^{-1} and $M = 50,000$. Comparison of $\mathcal{K}_R(t)$ shown in Fig. 6 of main text and $\mathcal{K}_I(t)$ shown in Fig. S1 confirms that these results all approach the values without the effect of bath in the short time limit.

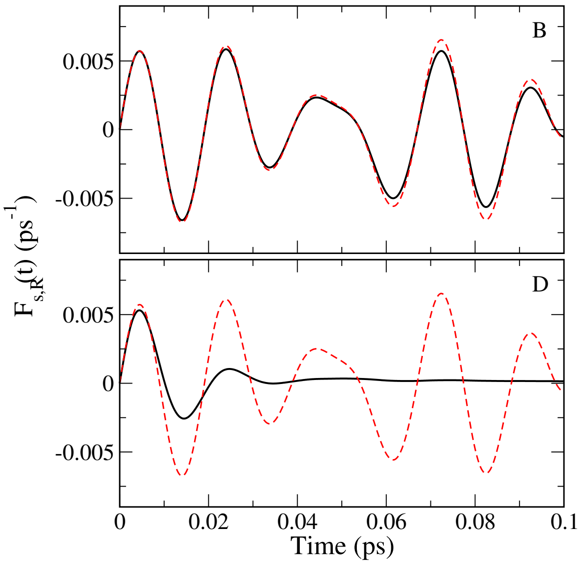

FIG. S4. Plots of $F_{s,R}(t) = F_R(t)/\hbar$ (in the unit of ps^{-1}) versus time for the two different cases B and D of Table 3 in the main text for azulene, shown as black lines. The data without bath are provided as red dashed lines for reference.

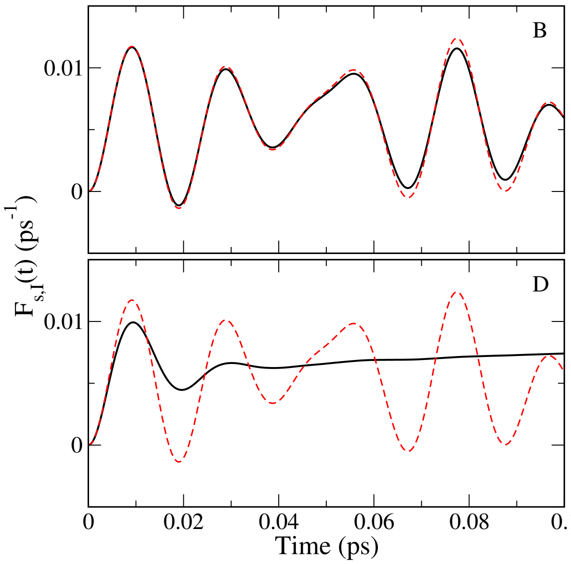

FIG. S5. Plots of $F_{s,I}(t) = F_I(t)/\hbar$ (in the unit of ps^{-1}) versus time for the two different cases B and D of Table 3 in the main text for azulene, shown as black lines. The data without bath are provided as red dashed lines for reference.

Similar approaches were used to decompose $\mathcal{J}_D(\omega)$ and $\mathcal{J}_F(\omega)$ into Ohmic parts and to calculate the contributions of the remainder as described above. Figures (S2)-(S5) show real and imaginary parts of the resulting $D(t)$ and $F(t)$ in the short time limit for the two cases B and D of Table 3 in the main text. These results all confirm that these correlation functions decay fast as the strength of bath increases (except for slow logarithmic increase for $F_I(t)$) whereas they all approach correct bath-free values in the short time limit.

[1]This assumption will be relaxed later once adiabatic basis of molecular states are identified.
[2]S. J. Jang, *Quantum Mechanics for Chemistry* (Springer Nature, New York, 2023).
[3]S. Jang, "Nonadiabatic quantum liouville equation and master equations in the adiabatic basis," J. Chem. Phys. **137**, 22A536 (2012).
[4]The original notations were slightly altered and $\hbar$ was incorporated into derivative coupling terms in this work.
[5]H. Hellmann, *Einführung in die Quantrnchemie* (Franz Deuticke, Leipzig, 1993).
[6]R. P. Feynman, "Forces in molecules," Phys. Rev. **56**, 340–343 (1939).
[7]C. Eckert, "Some studies concerning rotating axes and polyatomic molecules," Phys. Rev. **47**, 552–558 (1935).
[8]J. D. Louck and H. W. Galbraith, "Eckart vectors, eckart frames, and polyatomic molecules," Rerv. Mod. Phys. **48**, 69–106 (1976).
[9]H. M. Pickett and H. L. Strauss, "Conformational structure, energy, and inversion rates of cyclohexane and some related oxanes," J. Am. Chem. Soc. **92**, 7281–7290 (1970).
[10]A. Y. Dymarsky and K. N. Kudin, "Computation of the pseudorotation matrix to satisfy the eckart axis conditions," J. Chem. Phys. **122**, 124103 (2005).
[11]B. K. Min, D. Kim, D. Kim, and Y. M. Rhee, Bull. Kor. Chem. Soc. **44**, 989–1003 (2023).
[12]A. Garg, J. N. Onuchic, and V. Ambegaokar, ""Effect of friction on electron transfer in biomolecules"," J. Chem. Phys. **83**, 4491 (1985).
[13]More generally, this can be assumed to be different for each vibrational mode.
